# RAGE Signalling in Acute Inflammatory Disorders: Therapeutic Potential of Natural Products

**DOI:** 10.3390/biom16070929

**Published:** 2026-06-23

**Authors:** Qiqi Wang, Wenjuan Luo, Qihang Wan, Yuying Li, Diane Latawiec, Robert Sutton, John Windsor, Wei Huang, Peter Szatmary, Tingting Liu

**Affiliations:** 1 West China Centre of Excellence for Pancreatitis, Institute of Integrated Traditional Chinese and Western Medicine, West China Hospital, Sichuan University, Chengdu 610041, China; 2 Liverpool Pancreatitis Research Group, Institute of Systems, Molecular and Integrative Biology, University of Liverpool, Liverpool L69 3GE, UK; 3 West China School of Medicine, Sichuan University, Chengdu 610041, China; 4 Surgical and Translational Research Centre, Department of Surgery, Faculty of Medicine and Health Sciences, University of Auckland, Auckland 1142, New Zealand; 5 West China Biobank, West China Hospital, Sichuan University, Chengdu 610041, China; 6 Institutes for Systems Genetics, Frontiers Science Centre for Disease-Related Molecular Network, West China Hospital, Sichuan University, Chengdu 610200, China

**Keywords:** RAGE, acute inflammatory disorders, cytokine storm, phytotherapy, natural products

## Abstract

Acute inflammatory disorders, including acute lung injury, acute pancreatitis, ischaemia–reperfusion injury, and sepsis, are major clinical challenges characterised by rapid progression, a characteristic cytokine storm, and high mortality rates. The receptor for advanced glycation end-products (RAGE) serves as a pivotal multi-ligand pattern recognition receptor that integrates PAMPs and DAMPs. Excessive RAGE engagement triggers detrimental signalling cascades, notably NF-κB and MAPKs, which exacerbate hyperinflammation and lead to progressive organ dysfunction. Consequently, the RAGE axis represents a potent therapeutic target for mitigating hyperinflammation and improving clinical outcomes in acute inflammatory disorders. While initial pharmacological efforts focused on synthetic inhibitors and biologics, there is a shifting focus toward bioactive alternatives with high safety profiles. Here, we present recent molecular insights into RAGE-mediated pathogenesis in acute inflammatory disorders and evaluate current therapeutic strategies. Furthermore, we emphatically summarise the bioactive natural products, including terpenoids, flavonoids, alkaloids, and a xanthone, that prevent and treat acute inflammatory disorders by disrupting RAGE–ligand interactions and suppressing downstream oxidative stress and cytokine release. Integrating these molecular mechanisms with the pharmacological profiling of natural RAGE modulators provides a robust foundation for the development of next-generation therapeutic strategies to improve clinical outcomes in acute inflammatory disorders.

## 1. Introduction

Acute inflammatory disorders refer to diseases driven by a rapid, excessive, and uncontrolled inflammatory response, often triggered by infection or tissue injury, which can progress to systemic inflammation and multi-organ dysfunction syndrome (MODS). Acute lung injury or acute respiratory distress syndrome (ALI/ARDS) represents one of the most critical manifestations of these disorders, with a 35–45% hospital mortality among 459 intensive care units from 50 countries [1]. Acute pancreatitis is also an increasingly prevalent acute inflammatory disorder without specific therapy [2,3]. In severe cases, it can quickly evolve from sterile inflammation to ALI/ARDS within 24 h of hospital admission or sepsis-associated MODS during the advanced phase, the latter carrying a mortality rate above 50% [4,5,6]. Furthermore, ischaemia–reperfusion injury (IRI) in major organs such as the brain, heart, liver, and kidney shares similar inflammatory signatures [7]. Although initiated by distinct primary insults, these conditions converge on similar inflammatory cascades, frequently driven by endogenous danger signals [8,9,10,11]. Sepsis, a complex disorder responding to the infection, associated with acute organ dysfunction, influenced 166 million people and 21.4 million sepsis-related deaths annually, which represents approximately 31.5% of total global mortality [3,12]. Therefore, elucidating the molecular mechanisms underlying these responses is essential for improving severity prediction and developing novel therapeutic strategies.

Acute inflammatory response is typically initiated through the interaction between pathogen-associated molecular patterns (PAMPs) and pattern recognition receptors (PRRs) expressed on innate immune cells. Also, it is activated by damage-associated molecular patterns (DAMPs), which are released from stressed or injured cells in response to physical, chemical, or metabolic insults [13,14,15,16]. Upon pathogen invasion or tissue injury, PRRs such as Toll-like receptors (TLRs), which constitute the first line of host defence against infection, recognise their ligands and trigger a cascade of intracellular signalling events, leading to the production of proinflammatory cytokines and the onset of local acute inflammation. Cytokine-mediated inflammation helps suppress pathogens and activates the adaptive immune response to ultimately eliminate the infection [17]. However, excessive or dysregulated inflammation can be detrimental. When physiological homeostasis is disrupted, the acute inflammatory response may become prolonged and develop into acute inflammatory disorders [18].

Despite decades of research identifying TLRs as primary sensors of PAMPs and DAMPs, their inhibition alone is insufficient to quench the complex inflammatory cascade [19]. This underscores the necessity of investigating non-redundant pathways, most notably the receptor for advanced glycation end-products (RAGE), which functions as a PRR with an extensive ligand recognition and broad systemic expression [13]. These properties position RAGE as a key integrator of both endogenous and exogenous danger signals, thereby contributing to the pathogenesis of acute inflammatory disorders including ALI/ARDS, IRI, acute pancreatitis, and sepsis [20]. Given its pivotal role in amplifying the inflammatory responses, RAGE has attracted increasing attention as a promising therapeutic target for acute inflammatory disorders [13,21]. Moreover, RAGE knockout mice are viable and exhibit no major physiological abnormalities [22], suggesting that therapeutic modulation of RAGE-mediated signalling may represent an innovative strategy to mitigate hyperinflammatory states and improve outcomes in patients with acute and critical inflammatory conditions.

Despite considerable preclinical and early clinical investigation, no RAGE inhibitor has yet achieved successful Phase III clinical translation, highlighting the need to identify additional therapeutic candidates and alternative intervention strategies. In particular, natural products represent a valuable source of structurally diverse scaffolds with broad biological activities that can be directly developed or further optimised for drug discovery [23]. These properties suggest that natural products may represent promising candidates for the modulation of RAGE-associated inflammatory signalling in acute inflammatory disorders.

This review provides a comprehensive analysis of the structural characteristics, expression profiles, and ligand interactions of RAGE, alongside its downstream signalling effectors. Furthermore, we highlight recent progress in targeting the RAGE axis by discussing various therapeutic modalities, including small-molecule inhibitors, biologics, and natural compounds, while evaluating their potential as innovative strategies to improve clinical outcomes in acute inflammatory disorders.

## 2. RAGE: Structure, Expression, and Isoforms

**Figure 1:**
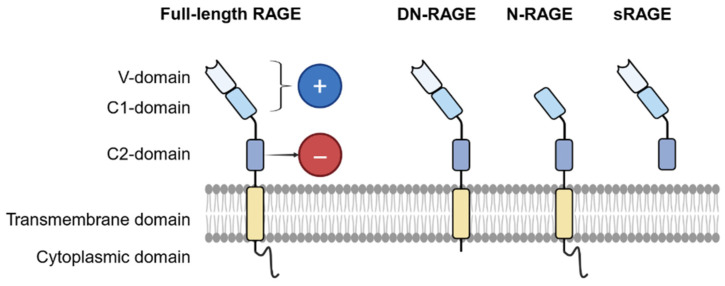
**Domain architecture and isoforms of RAGE.** RAGE consists of an extracellular ligand-binding domain, a transmembrane helix, and a cytoplasmic tail. The blue ‘+’ and red ‘−’ circles represent positive and negative charges, respectively. DN-RAGE lacks the cytoplasmic tail and can bind ligands without initiating downstream signalling. N-RAGE lacks the V-domain, thereby limiting its ability to bind certain ligands. sRAGE acts as a decoy receptor by sequestering ligands without transducing signals.

RAGE is a 50–55 kDa type I transmembrane protein belonging to the immunoglobulin superfamily, first identified in 1992 [24]. Its gene is located on chromosome 6 near the major histocompatibility complex class II region, linking it to immune and inflammatory processes [25,26]. Structurally (Figure 1), RAGE consists of an extracellular ligand-binding domain, a transmembrane helix, and a cytoplasmic tail [27,28]. The extracellular domain includes the V (residues 23–116), C1 (residues 124–221), and C2 (residues 227–317) domains [21,28,29,30]. The V and C1 domains together form the VC1 unit, which provides a highly positively charged surface that serves as the primary interaction site for negatively charged ligands [21,26,29,31]. While the cytoplasmic tail (residues 363–404) is essential for intracellular signalling, the transmembrane domain (residues 343–363) is thought to mediate receptor dimerisation or clustering, both of which are required for signal transduction [32]. Furthermore, RAGE multimerisation on the cell surface facilitates ligand binding and is critical for inflammatory responses [29,33]. Despite extensive research on the extracellular domain, the dynamic regulation of the transmembrane and cytoplasmic domain-mediated events requires deeper mechanistic exploration [29,31,34].

Under physiological conditions, RAGE is highly expressed in the lungs, particularly in alveolar epithelial cells, endothelial cells, and macrophages [35,36,37,38,39]. In contrast, it is maintained at moderate levels in other tissues such as the brain, heart, liver, kidney, vasculature, and muscles [40,41,42,43,44]. During inflammation, however, RAGE expression is significantly upregulated through NF-κB activation, creating a self-perpetuating cycle that amplifies the inflammatory cascade [45,46].

Alternative splicing and proteolytic cleavage generate several RAGE isoforms with distinct function roles. Full-length RAGE (residues 23–404) facilitates downstream signalling associated with inflammation, oxidative stress, proliferation, migration, and apoptosis [32,47,48,49,50]. These effects are often reinforced through positive feedback mechanisms that enhance both receptor expression and ligand production [51,52,53,54,55]. Conversely, dominant-negative RAGE (residues 23–363) lacks the cytoplasmic tail, allowing it to bind ligands without initiating signalling transduction, thereby dampening inflammation [29,54,56,57]. N-terminal truncated RAGE (N-RAGE, residues 124–404) lacks the V-domain, preventing ligand binding but potentially activating alternative signalling pathways [28,49,58,59]. Finally, soluble RAGE (sRAGE, residues 23–342), generated by proteolysis or splicing, functions as a decoy receptor, sequestering ligands without signalling transduction [60,61,62,63]. sRAGE is a potential biomarker for diseases such as cardiovascular and neurodegenerative diseases [60,61,62,64], with therapeutic potential in reversing RAGE-mediated pathological effects [52,65,66,67].

## 3. RAGE Ligands and Effects in Acute Inflammatory Disorders

Owing to its broad expression and ligand-binding versatility, RAGE mediates a range of pathological signal transduction events under disease conditions. While previous reviews have summarised the involvement of RAGE in the pathophysiological processes of diabetes, neurodegenerative disorders, cardiovascular diseases, cancer, and other chronic conditions, its specific contributions to acute inflammatory disorders warrant a more systematic characterization [13,68]. In acute inflammatory settings, the interaction between RAGE and its diverse ligands amplifies proinflammatory pathways and drives cellular responses that exacerbate disease progression (Table 1).

Lipopolysaccharide (LPS), a key component of Gram-negative bacteria outer membranes, exemplifies this mechanism [69]. Beyond its classical TLR4-mediated effects, LPS directly binds RAGE, activating NF-κB independently to promote cytokine production such as TNF-α and IL-6, as well as endothelial hyperpermeability, contributing to the progression of septic shock and organ dysfunction [13,69,86].

High mobility group box 1 (HMGB1), released during cellular stress or necrosis, further amplifies inflammation through RAGE [87,88]. HMGB1 binds RAGE via residues 23–50 and 150–183, activating ERK1/2, p38 MAPK, and NF-κB to drive cytokine release, dendritic cell maturation, as well as inflammasome-mediated pyroptosis in ALI/ARDS and sepsis [68,70,89,90,91,92,93,94]. Anti-HMGB1 antibodies improve survival in preclinical sepsis models by attenuating neutrophil recruitment and vascular leakage, highlighting its therapeutic potential [94,95,96,97,98].

Closely linked to the role of HMGB1, S100 proteins, particularly S100A8/A9 and S100A12 (extracellular RAGE-binding protein, EN-RAGE), act as DAMPs that bind RAGE to activate PI3K/AKT/mTOR, NF-κB, and ERK pathways [75,94,99,100,101,102,103]. S100B oligomerisation enhances RAGE-dependent ERK and NF-κB signalling, underscoring the functional diversity of S100-RAGE interactions [34,72,104,105,106]. Inhibitors targeting S100-RAGE reduce inflammation in experimental autoimmune models, demonstrating translational relevance [107].

Extracellular deoxyribonucleic acid (DNA), derived from host or pathogen sources, synergises with HMGB1 to form complexes that bind RAGE. This interaction facilitates RAGE-mediated endosomal trafficking of DNA, amplifying NF-κB-driven cytokine production [13,77,108,109]. Similarly, complement components like C3a and C1q interact with RAGE to modulate phagocytosis and inflammation. C3a-RAGE binding forms ternary complexes with oligonucleotides [110], while multivalent C1q-RAGE interactions enhance monocyte phagocytosis, bridging complement activation to RAGE-mediated immune responses [78,111].

Heat shock protein 70 (HSP70) further diversifies the roles of RAGE. Extracellular HSP70 binds to oligomeric RAGE via disulphide bonds, activating ERK1/2 and NF-κB to upregulate TNF-α, IL-1β, and IL-6 [79]. This establishes a proinflammatory feedback loop in oxidative stress models, while in cancer contexts, HSP70-RAGE binding may paradoxically promote immunosuppressive microenvironments [80].

Advanced glycation end-products (AGEs), generated via Maillard reactions under hyperglycaemia or oxidative stress, bind RAGE to activate MAPK, JAK/STAT, and NF-κB pathways [70,94,112,113,114]. This drives reactive oxygen species (ROS) production, glutathione depletion, and cytokine release, thereby exacerbating cellular damage and prolonging inflammation [115,116,117,118]. sRAGE acts as a decoy receptor, mitigating AGE-induced damage in preclinical studies, though physiological glycation levels may attenuate its inflammatory potential [119,120,121].

Phosphatidylserine, exposed on apoptotic cell surfaces, engages RAGE to activate mDia1/Rac1 pathways, mediating phagocytic clearance [82,94,122,123]. RAGE deficiency impairs neutrophil clearance, leading to unresolved inflammation. Phosphatidylserine–RAGE interactions are vital for maintaining tissue homeostasis [85,122].

**Figure 2:**
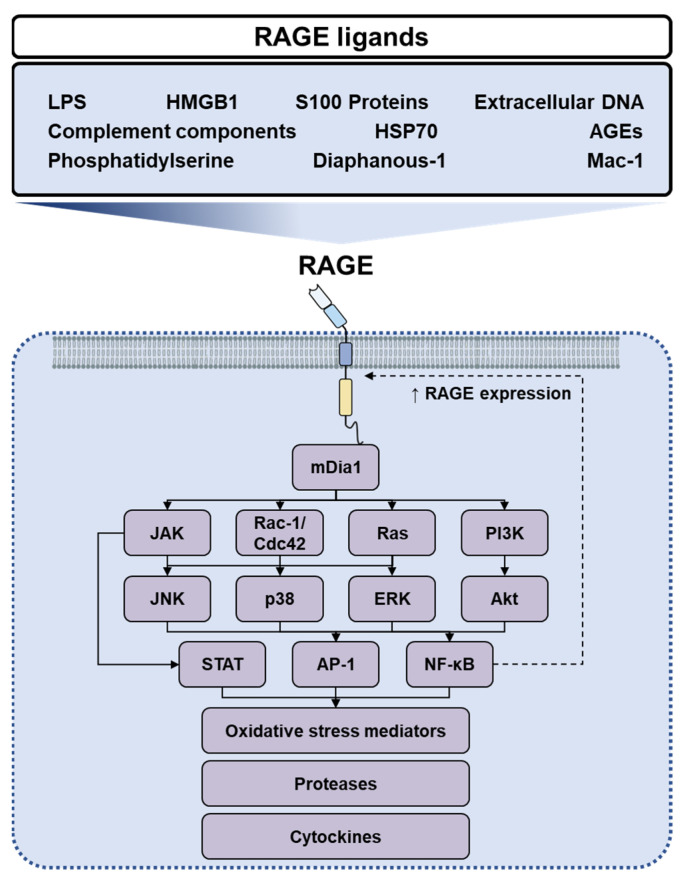
**RAGE ligands and downstream signalling pathways.** Upon ligand binding, RAGE activates multiple signalling pathways, leading to changes in gene expression and altered cellular functions, including oxidative stress, inflammation, and the upregulation of RAGE expression itself.

These RAGE ligands help to orchestrate acute inflammation through interconnected pathways, spanning pathogen detection (LPS, DNA), DAMPs (HMGB1, S100), phagocytosis (phosphatidylserine), and leukocyte recruitment (integrins). Upon ligand engagement, RAGE converges on core downstream signalling cascades. NF-κB is universally activated across all ligand classes, driving sustained proinflammatory gene expression; MAPK pathways (ERK1/2, p38) are predominantly triggered by HMGB1, S100 proteins, HSP70, and AGEs to regulate stress responses and cytokine amplification; PI3K/AKT/mTOR signalling is selectively induced by S100 proteins, enhancing cellular survival and metabolic reprogramming; Rho GTPases (including mDia1 and Rac1) mediate cytoskeletal dynamics for phagocytosis and migration upon phosphatidylserine binding, while JAK/STAT activation is primarily linked to AGEs, further amplifying immune cell activation (Figure 2). Therapeutic strategies targeting specific ligand–RAGE interactions show promise, though context-dependent effects will likely require precise intervention to preserve immune homeostasis.

## 4. Pharmacological Inhibition of RAGE in Acute Inflammatory Disorders

The interaction between its various ligands plays a significant role in the pathogenesis of acute inflammatory disorders [13,21,124,125]. Elevated plasma levels of sRAGE were observed following severe trauma and surgical procedures, correlating with injury severity and prognosis [126,127]. Furthermore, most prospective clinical studies have shown that circulating sRAGE levels are closely linked to the severity and mortality of ALI/ARDS, acute pancreatitis, and sepsis (Table 2). These clinical findings are mirrored in animal models, where RAGE expression is markedly increased, and genetic RAGE knockout models have consistently demonstrated protective effects [128,129,130,131,132,133].

### 4.1. Targeting RAGE with Synthetic Small Molecules and Biologics

Given its central role in amplifying inflammation, RAGE has emerged as an attractive therapeutic target. Over the past two decades, in addition to sRAGE, which can act as a decoy receptor as mentioned earlier, various drugs targeting RAGE have been developed, including small-molecule compounds, RAGE antibodies, and biologics (Table 3). These drugs target different RAGE domains and have been evaluated across multiple disease models.

Azeliragon (TTP488, also referred to as PF-04494700) is an orally administered small-molecule RAGE inhibitor capable of crossing the blood–brain barrier (BBB) [158]. It exhibits high specificity and affinity for RAGE, with a reported dissociation constant of 12.7 ± 7.6 nM for recombinant human sRAGE [174]. Preclinical studies in Alzheimer’s disease (AD) animal models demonstrated that azeliragon reduced amyloid-β (Aβ) plaque deposition and decreased total Aβ concentrations in the brain, while concomitantly increasing plasma Aβ levels [175]. To date, azeliragon is the only RAGE inhibitor that has advanced into clinical trials. A clinical study in patients with mild to moderate AD showed that azeliragon was safe and well tolerated after 10 weeks of treatment, although no consistent effects were observed on plasma β-amyloid levels, inflammatory biomarkers, or secondary cognitive outcomes [158]. A subsequent secondary analysis of a phase 2B trial in patients with mild AD revealed a significant reduction in ADAS-Cog scores at 18 months, along with a trend toward reduced CDR-SB and ADCS-ADL scores [176]. While extensively studied in AD, azeliragon has been shown to exert anti-inflammatory effects in acute lung injury by targeting RAGE-mediated pathways. In an LPS-induced mouse model, azeliragon reduced neutrophil-driven airway inflammation and lung injury, lowered IL-6, IL-1β, and TNF-α levels in bronchoalveolar lavage fluid (BALF), and mitigated alveolar–capillary barrier disruption and pulmonary oedema [157].

FPS-ZM1 was initially identified to block the binding of Aβ to the V domain of RAGE and readily crosses the BBB. FPS-ZM1 inhibits β-secretase activity and Aβ production by specifically binding to RAGE in brain tissue and concurrently suppresses microglial activation and neuroinflammatory responses. In an aged AD mouse model, inhibition of RAGE activity at the BBB and within brain tissue by FPS-ZM1 significantly reduced Aβ40 and Aβ42 levels, restored cognitive function, and normalised cerebral blood flow responses [48]. As the most widely used RAGE inhibitor in preclinical studies, FPS-ZM1 has also been extensively evaluated in multiple models of acute inflammatory disorders. In an LPS-induced acute lung injury mouse model, FPS-ZM1, similar to azeliragon, improved airway epithelial barrier dysfunction by reducing the expression of RAGE and its ligands [157]. In a caecal ligation and puncture (CLP)-induced ARDS mouse model, FPS-ZM1 alleviated lung inflammatory injury and cell apoptosis, accompanied by downregulation of HMGB1, RAGE, and endoplasmic reticulum stress-related proteins [159]. Furthermore, inhibition of HMGB1–RAGE interactions by FPS-ZM1 markedly attenuated limb IRI-induced vascular damage in rats [160]. In a diabetic-obese mouse myocardial IRI model, FPS-ZM1 significantly reduced infarct size and decreased serum lactate dehydrogenase and CK-MB levels [161]. FPS-ZM1 also exerted protective effects in CLP-induced sepsis-associated acute brain injury [162].

Anti-RAGE antibodies (abRAGEs) have also demonstrated protective effects in several sepsis-related animal models. In an LPS-induced neonatal rat ALI model, abRAGE treatment reduced the expression of RAGE and NF-κB in lung tissue, alleviated histopathological abnormalities, and significantly improved lung injury scores [55]. In a galactosamine and LPS-induced acute liver injury model in mice, abRAGE mitigated hepatic microcirculatory deterioration and leukocyte recruitment, thereby attenuating liver damage [164]. Moreover, in a CLP-induced sepsis model in mice, blockade of RAGE–ligand interactions using abRAGE markedly improved survival rates [163].

Additionally, a synthetic peptide corresponding to the HMGB1 COOH-terminal motif (amino acids 150–183) effectively inhibited metastatic foci formation in an experimental pulmonary metastasis model by disrupting amphoterin–RAGE interactions [165]. A subsequent study further demonstrated that this peptide downregulated RAGE expression in lung tissue in an ALI animal model, reduced the levels of TNF-α, IL-6, and IL-1β in BALF and lung tissue, and markedly attenuated pulmonary inflammatory responses [166].

Several other inhibitors show promise but await rigorous testing in acute inflammatory disorders. GM-1111 has been shown to inhibit the interaction between RAGE and multiple ligands, including AGE, S100B, and HMGB1, thereby exerting anti-inflammatory activity [167]. Similarly, an S100P-derived peptide effectively blocks the interaction of S100P, S100A4, and HMGB1 with RAGE, leading to reduced NF-κB activation and inhibition of tumour growth and metastasis in experimental cancer models [168]. 4,6-bis(4-chlorophenyl)pyrimidine analogues directly bind to RAGE, markedly reduce the levels of soluble Aβ in the brain, and improve cognitive function in an AD mouse model [169]. Pyrazole-5-carboxamide derivatives similarly inhibit RAGE activity and decrease brain amyloid-β levels in experimental models [170]. In addition, an aptamer-based RAGE antagonist disrupts the interaction between RAGE and S100B, thereby blocking downstream NF-κB signalling and suppressing tumour growth and microvascular formation in colorectal cancer models [171]. Furthermore, a group of 13 small-molecule compounds were identified as competitive inhibitors of the interaction between the cytoplasmic tail of RAGE and diaphanous-related formin 1, blocking RAGE ligand-dependent stimulation and demonstrating therapeutic potential in RAGE-associated diseases [172]. ZINC000003844291 and ZINC000031829829 were also reported to exhibit high binding affinity to the catalytic region of the V domain, representing promising drug-like candidates for future development [173].

### 4.2. Natural Products as Inhibitors of the RAGE Signalling Axis

**Figure 3:**
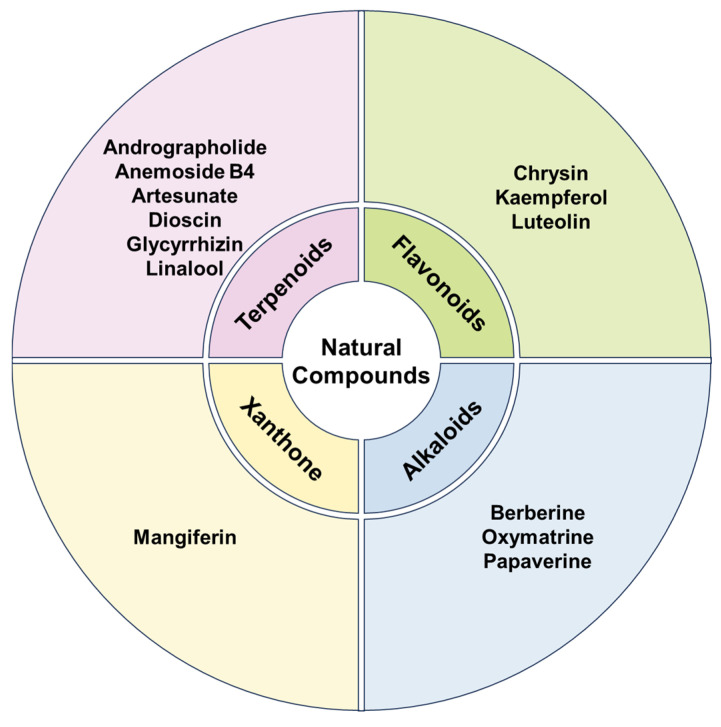
**Classification of RAGE-targeting natural compounds.** Natural compounds derived from CHM formulations are classified into terpenoids, flavonoids, alkaloids, and a xanthone. These compounds ameliorate various acute inflammatory disorders by targeting RAGE–ligand interactions and downstream signalling pathways.

Complementing synthetic pharmacological interventions, accumulating evidence demonstrates that various natural compounds can modulate RAGE-associated signalling pathways in preclinical models of acute inflammatory disorders (Figure 3), of which the specific mechanisms and therapeutic outcomes have been evaluated across a diverse range of rodent models (Table 4).

Dioscin, a natural steroid saponin, alleviated cerebral damage in both in vitro and in vivo rat IRI models by downregulating the HMGB1/RAGE signalling pathway, reducing TNF-α, IL-6, and ROS levels, and enhancing antioxidant defences (superoxide dismutase, glutathione) [177].

Mangiferin [178] and kaempferol [179] each inhibited AGE-RAGE signalling and modulated MAPK pathways to reduce oxidative stress, inflammation, and apoptosis, thereby preserving cardiac function in diabetic myocardial IRI.

Chrysin [180], acting as a PPAR-γ/Nrf2 agonist, suppressed AGE-RAGE/NF-κB signalling and enhanced antioxidant defences in diabetic hearts, whereas artesunate [181], alone or in combination with rapamycin, protected against hepatic IRI by inhibiting HMGB1/RAGE and TLR4/MyD88/TRAF6/NF-κB pathways.

Linalool also ameliorated hepatic IRI by activating the Keap1/Nrf2/HO-1/NQO-1 antioxidant pathway and suppressing the HMGB1/TLR4/RAGE/NF-κB cascade [182].

Luteolin, a natural flavonoid, protects against LPS-induced hepatic injury by modulating the P2X7R-RAGE-TLR4 axis, thereby suppressing HMGB1 release and mitigating inflammatory responses in the liver [183].

Anemoside B4, a major bioactive saponin isolated from the roots of Pulsatilla chinensis, exhibits potent anti-inflammatory and antioxidant activities. In a CLP-induced ALI mouse model, anemoside B4 significantly attenuated lung injury by modulating the AGE/RAGE–Nrf2 signalling axis, thereby suppressing ferroptosis and inflammatory responses [191].

Andrographolide, a diterpene lactone from Andrographis paniculata, was shown in CLP-induced sepsis-associated ALI models and LPS-stimulated macrophage cells to promote autophagy in alveolar macrophages by binding to RAGE and inhibiting the PI3K/AKT/mTOR pathway. This action suppressed NLRP3 inflammasome activation and reduced proinflammatory cytokine release, thereby ameliorating the severity of ALI [190].

Glycyrrhizin, a triterpene glycoside from liquorice, was found in CLP-induced sepsis and in LPS- or HMGB1-stimulated macrophages to block the interaction of HMGB1 with RAGE and TLR4, resulting in inhibition of the downstream MAPK/NF-κB pathway and a consequent reduction in inflammatory cytokine production [184].

Berberine has been reported to improve cognitive impairment in sepsis-associated encephalopathy by targeting the HMGB1/RAGE axis, reducing hippocampal inflammatory mediators, and protecting against microglia-induced astrocyte activation [188].

Oxymatrine, derived from Sophora flavescens, improved outcomes in CLP-induced sepsis models by inhibiting HMGB1/RAGE/NF-κB signalling, thereby attenuating cytokine production and organ injury [189].

Papaverine has been shown in sepsis models to inhibit the HMGB1/RAGE interaction by increasing sRAGE levels, thus reducing proinflammatory cytokine release, oxidative stress, and tissue damage [185,186,187].

Despite these encouraging preclinical findings, there is currently no registered or published clinical trial worldwide evaluating natural compounds as RAGE inhibitors for acute inflammatory disorders, and no published clinical studies have demonstrated the therapeutic efficacy of natural-product-mediated RAGE inhibition in human acute inflammatory disorders. Therefore, the clinical translation of natural products remains at a very early stage.

Most evidence is still restricted to cellular and rodent models. A key limitation relates to pharmacokinetic properties, as many natural compounds exhibit poor aqueous solubility and low bioavailability, which reduce in vivo efficacy [192]. In addition, only a limited number of studies have investigated direct binding interactions between natural compounds and RAGE, resulting in incomplete mechanistic understanding, insufficient selective target validation, and limited rational optimisation of clinical candidates. Moreover, the multitarget properties of many natural compounds may obscure the specific contribution of RAGE inhibition to observed therapeutic effects, hindering mechanism-based clinical development. From a translational and manufacturing perspective, most small-molecule natural products discussed here can be chemically synthesised or semi-synthetically produced. However, for complex saponins such as dioscin and anemoside B4, scalable and cost-effective industrial synthesis remains challenging, and production still relies on plant extraction or semi-synthetic approaches, which may limit batch standardisation and large-scale development.

Several approaches may facilitate further development of natural products. Structural optimisation and semi-synthetic modification may improve pharmacokinetic and pharmacodynamic properties [193]. Drug-delivery systems, such as nanoparticles, liposomes, and prodrug strategies, may enhance bioavailability and tissue targeting [192,194]. Moreover, integrating computational modelling, molecular docking, and structure–activity relationship analyses with experimental validation may improve the identification of selective and potent RAGE inhibitors and provide mechanistic insight into ligand–RAGE interactions. Standardised extraction protocols and rigorous quality-control procedures are also required to ensure reproducibility and consistency for plant-derived agents. Finally, well-designed preclinical studies in experimental models and biomarker-guided precision designs are needed to clarify mechanisms, evaluate safety and efficacy, and reduce biological heterogeneity. Collectively, these advances may facilitate the development of natural-product RAGE inhibitors for acute inflammatory disorders.

## 5. Conclusions

The pathogenesis of acute inflammatory disorders, encompassing life-threatening conditions such as ALI/ARDS, acute pancreatitis, IRI, and sepsis, is characterised by a rapid and uncontrolled convergence of inflammatory cascades. As established in this review, RAGE functions as a non-redundant and indispensable regulator in the pathogenesis of acute inflammatory disorders. By interacting with diverse ligands, including HMGB1, S100 proteins, and AGE, RAGE acts as a central molecular bridge by activating downstream pathways such as NF-κB and MAPKs, thereby promoting excessive cytokine release, tissue injury, and organ dysfunction that translates initial infectious or sterile insults into sustained systemic inflammation.

RAGE knockout models suggest that the receptor is not essential for normal physiological homeostasis, thereby positioning it as a potentially safe therapeutic target. Translating the mechanistic understanding of the RAGE axis into clinical efficacy has led to the exploration of diverse therapeutic strategies. This review has systematically detailed the potential of sRAGE as a decoy receptor, synthetic small-molecule inhibitors such as azeliragon and FPS-ZM1, and targeted biologics. Concurrently, natural compounds, including terpenoids, flavonoids, alkaloids, and a xanthone, have emerged as significant modulators capable of quenching hyperinflammatory states across various preclinical rodent models by modulating RAGE-associated signalling pathways. These interventions offer a promising avenue to address the limitations of TLR-centric therapies, providing a supplemental approach to inhibiting the complex inflammatory signalling networks that drive acute organ injury.

Despite these promising therapeutic landscapes, a notable translational gap remains. Most synthetic inhibitors have been evaluated primarily in chronic diseases, with no agents successfully advancing through Phase III clinical trials. In this context, increasing attention has been directed toward natural products in preclinical models of acute inflammatory disorders. However, the evidence supporting the efficacy of natural products derives almost exclusively from in vivo and in vitro experimental models, and no clinical trials in humans have yet demonstrated their therapeutic efficacy in acute inflammatory disorders. Crucially, for these natural products to move beyond preclinical observation, future research must elucidate their precise binding kinetics with the RAGE. Furthermore, addressing the inherent pharmacokinetic limitations of these compounds, particularly low solubility and poor bioavailability, through innovative drug delivery systems or structural derivatives will be essential for achieving therapeutic concentrations in inflamed organs.

In conclusion, the available evidence supports the status of RAGE as a clinically relevant and druggable target for mitigating the excessive inflammatory responses that characterise acute disorder states. Natural compounds, with their diverse chemical scaffolds, represent a vital resource for the development of next-generation RAGE antagonists. Advancing these strategies through refined structural studies, rigorous mechanistic studies and well-designed clinical trials will be essential to improving therapeutic outcomes, ultimately fulfilling the urgent need for novel interventions in the management of acute and critical inflammatory conditions.

## Figures and Tables

**Table 1:** **RAGE** **ligands** **in** **inflammatory** **disorders.**

Ligands	Binding Domain	Downstream Activation	Effects	References
LPS	V	NF-κB	Accelerating acute inflammatory reactions and vascular dysregulation	[69]
HMGB1	VC1C2	ERK1/2 p38 MAPK PKCbII	Promoting immune cell maturation, activation, and cytokine production	[70,71,72]
S100 Proteins	VC1 or C1 and/or C2	ERK MEK MAPK NF-κB AKT JNK	Activating transcription of pro-inflammatory factors and leading to ROS production and apoptosis	[73,74,75,76]
Extracellular DNA	VC1	NF-κB	Promoting inflammatory responses	[77]
Complement Components	N/A	N/A	Enhancing phagocytosis	[78]
HSP70	N/A	ERK1/2 NF-κB	Increasing secretion of TNF-α and phagocytosis	[79,80]
AGEs	V	MAPK TGF-β NF-κB	Activating downstream signalling pathways leading to increased ROS production and inflammatory cytokines	[27,81]
Phosphatidylserine	N/A	Rac1/Cdc42 ERK	Triggering the phagocytic process	[82,83]
Diaphanous-1	Cytoplasmic domain	Rac1 Cdc42	Signal transduction and cellular migration	[47]
Mac-1	N/A	N/A	Mediating leukocyte recruitment	[84,85]

LPS, lipopolysaccharide; NF-κB, nuclear factor-kappa B; HMGB1, high mobility group box 1; ERK, extracellular signal-regulated kinase; MAPK, mitogen-activated protein kinase; PKCbII, protein kinase C beta II; MEK, mitogen-activated protein kinase kinase; JNK, c-Jun N-terminal kinase; ROS, reactive oxygen species; DNA, deoxyribonucleic acid; N/A, not available; HSP70, heat shock protein 70; TNF-α, tumour necrosis factor alpha; AGEs, advanced glycation end-products; TGF-β, transforming growth factor beta; Rac1, ras-related C3 botulinum toxin substrate 1; Cdc42, cell division control protein 42 homolog; Mac-1, macrophage-1 antigen.

**Table 2:** **Clinical** **studies** **of** **sRAGE** **levels** **in** **acute** **inflammatory** **disorders.**

Diseases	Study Design	Measurement	Time Points	Correlations	References
ALI/ARDS	RCT *	sRAGE levels in plasma	>Day 0 >Day 3	Baseline plasma RAGE levels are strongly associated with clinical outcomes in patients with acute lung injury ventilated with higher tidal volumes	[134]
ALI/ARDS	Prospective observational study	sRAGE levels in plasma	>Day 2 >Every 3 days for the first month >Then once a week	In patients with ALI/ARDS, plasma sRAGE levels are elevated on day 1 and decrease over time	[135]
ALI/ARDS	Prospective observational study	sRAGE levels in plasma	>Day 0 >Day 3 >Day 6 >Day 28	Levels of sRAGE were correlated with ALI/ARDS severity and decreased over time but were not associated with outcome	[136]
ALI/ARDS	Prospective observational study	sRAGE levels in plasma	33 ± 39 h after intubation	Levels of sRAGE are associated with lung injury severity	[137]
ALI/ARDS	Prospective observational study	sRAGE and esRAGE levels in plasma	>ICU admission >Day 1	Plasma sRAGE, when measured upon ICU admission or on day one, could predict the development of ARDS within seven days	[138]
ALI/ARDS	Prospective observational study	sRAGE levels in serum	>Admission >24 h >72 h	Patients with acute respiratory failure requiring mechanical ventilation did not have a consistent change in sRAGE levels	[139]
ALI/ARDS	RCT **	sRAGE levels in plasma	>Day 0 >Day 1 >Day 2 >Day 3 >Day 4 >Day 6	>Association between changes in plasma sRAGE and the risk of death at day 90 >The association between the rate of change in sRAGE over time and 90-day survival was increased with higher values of baseline sRAGE	[140]
Acute pancreatitis	Prospective observational study	sRAGE levels in plasma	>On admission (within 72 h after the onset) >Day 1 >Day 2 >Day 3 >Day 4 >Day 7 >Day 12	>Levels of sRAGE positively associated with organ failure on admission and increased to the peak by days 3–4 only in the organ failure group >The maximum sRAGE levels were significantly higher in non-survivors	[141]
Acute pancreatitis	Prospective observational study	sRAGE levels in plasma	On admission (within 48 h after the onset)	The sRAGE concentrations in patients with severe acute pancreatitis was significantly lower than in patients with mild pancreatitis or that of controls	[142]
Acute pancreatitis	Prospective observational study	sRAGE levels in serum	On admission (within 72 h after the onset)	>Serum levels of sRAGE were significantly higher in patients with severe acute pancreatitis than in those with mild acute pancreatitis or moderate severe acute pancreatitis and healthy controls >The predictive ability of sRAGE for severe acute pancreatitis was comparable to that of the traditional indicators such as APACHE II score, CTSI and CRP >Serum sRAGE level was correlated with renal failure at admission, and with both renal and respiratory failure 1–7 days after admission >It could distinguish between transient (<48 h) and persistent (>48 h) organ failure	[143]
Sepsis	Prospective observational study	sRAGE levels in plasma	the first 24 h after onset of severe sepsis or septic shock	>Plasma sRAGE concentrations were higher in septic patients than in healthy volunteers >Within 24 h of the onset of sepsis, plasma sRAGE concentrations of nonsurvivors were significantly elevated compared with survivors	[144]
Sepsis-induced AKI	Prospective observational study	sRAGE levels in serum	at baseline	Increased serum level of RAGE compared with controls	[145]
CAP-associated sepsis	Prospective observational study	sRAGE levels in plasma	at enrolment	>Plasma sRAGE levels are elevated in CAP patients >sRAGE performed as an independent factor affecting the probability of a fatal outcome	[146]
Sepsis	Prospective observational study	sRAGE levels in serum	>Day 0 >Day 3	sRAGE levels do not change in the circulation of patients with sepsis	[147]
Sepsis	Prospective observational study	sRAGE levels in plasma	>Day 1 >Day 3	>Increased sRAGE was associated with 28-day mortality in patients with sepsis >sRAGE was increased in patients with individual organ failure	[148]
Sepsis	Prospective observational study	sRAGE levels in plasma	N/A	>Increased levels of sRAGE are associated with worse outcomes in patients with septic shock >Reduced sRAGE levels are associated with increased mortality	[149]
Sepsis	Cross-sectional study	sRAGE levels in plasma	Within 24 h after the diagnosis of severe sepsis	>The serum level of sRAGE was increased in severe sepsis patients compared with that in healthy volunteers >The serum level of IL-6 correlated positively with that of sRAGE >The serum level of sRAGE was significantly associated with that of sVCAM-1, an early marker of endothelial activation related to systemic inflammation	[150]
Sepsis	Prospective observational study	sRAGE levels in plasma	>Sepsis onset >24 h >Day 4 >Day 7 >Day 14 >Day 28	In patients with septic shock, sRAGE plasma levels were significantly increased	[151]
Sepsis	Prospective observational study	sRAGE levels in serum	Day 1 to 7	>The serum level of sRAGE was significantly higher in ICU patients compared with healthy controls >sRAGE did not predict mortality	[152]
Sepsis	Prospective observational study	sRAGE levels in plasma	ICU admission	No obvious association between circulating levels of sRAGE and clinical and biological indices that are usually recorded upon ICU admission	[153]
Sepsis	Prospective observational study	sRAGE levels in plasma	>Day 1 >Day 3 >Day 5	>sRAGE levels on day 1 (sRAGE 1) correlated with early inflammatory markers (TNF-α, IL-6, and IL-8) >Association between sRAGE 1 and 28-day mortality, with higher levels in non-survivors >The higher the sRAGE 1, the greater the decrease in plasma AGEs	[154]
Sepsis-Induced ARDS	Prospective observational study	sRAGE levels in plasma	>Day 1 >Day 2 >Day 3	Among critically ill patients with sepsis, sRAGE levels were not significantly different between ARDS and non-ARDS patients but were higher in nonsurvivors compared with survivors.	[155]

ALI/ARDS, acute lung injury or acute respiratory distress syndrome; RCT, randomised controlled trial; sRAGE, soluble RAGE; esRAGE, endogenous secretory RAGE; ICU, intensive care unit; APACHE II, Acute Physiology and Chronic Health Evaluation II; CTSI, computed tomography severity index; CRP, C-reactive protein; CAP, community-acquired pneumonia; N/A, not available; IL-6, interleukin-6; sVCAM-1, soluble vascular cell adhesion molecule-1; TNF-α, tumour necrosis factor alpha; AGEs, advanced glycation end-products. *, an RCT of lower tidal volume ventilation in ALI; **, a secondary analysis of the Lung Imaging for Ventilator Setting in ARDS (LIVE) multicentre RCT.

**Table 3:** **Synthetic** **small** **molecules** **and** **biologics.**

Inhibitors	Targeting of RAGE Domain	Effects	Application in Acute Inflammatory Disorders	References
Azeliragon/TTP488/PF-04494700	V	Inhibition of RAGE binding to its ligands	ALI	[156,157,158]
FPS-ZM1	V	Inhibition of RAGE binding to its ligands	ALI/ARDS IRI Sepsis	[48,157,159,160,161,162]
Anti-RAGE antibodies	N-terminal region	Inhibition of RAGE binding to its ligands	ALI ACLI Sepsis	[55,163,164]
HMGB1 (150–183) peptide	VC1C2	Inhibition of the HMGB1–RAGE interaction	ALI	[165,166]
GM-1111	VC1C2	Inhibition of RAGE binding to its ligands	None	[167]
S100P-derived peptide	VC1C2	Inhibition of RAGE binding to S100P, S100A4, and HMGB-1	None	[168]
4,6-bis(4-chlorophenyl)pyrimidine analogues	V	Inhibition of the Aβ-RAGE interaction	None	[169]
Pyrazole-5-carboxamides	V	Inhibition of the Aβ-RAGE interaction	None	[170]
Aptamer-based antagonist	V	Inhibition of the S100B-ctRAGE interaction	None	[171]
13 small molecule compounds	Cytoplasmic region	Inhibition of the mDia1-ctRAGE interaction	None	[172]
ZINC000003844291 ZINC000031829829	V	Inhibition of the S100A6-RAGE interaction	None	[173]

RAGE, receptor for advanced glycation end-products; ALI/ARDS, acute lung injury or acute respiratory distress syndrome; IRI, ischaemia–reperfusion injury; ACLI, acute liver injury; HMGB1, high mobility group box-1; Aβ, amyloid-β; ctRAGE, C-terminal RAGE; mDia1, mammalian diaphanous-related formin 1.

**Table 4:** **Natural** **compounds** **inhibiting** **RAGE-associated** **signalling** **pathways**.

Compounds	CID	2D Structure	Binding Score of Molecular Docking	Targets	Experimental Models	Treatment Regimen	Effects	References
Dioscin	119245	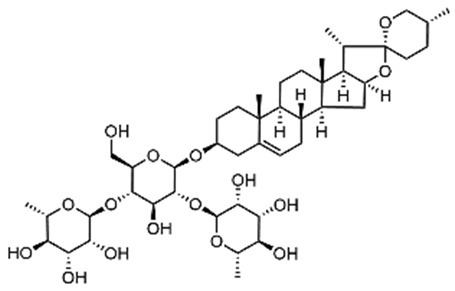	N/A	HMGB1-RAGE pathway	In vivo: MCAO/R-induced cerebral IRI in male Sprague-Dawley rats In vitro: OGD/R model in primary hippocampal neurons	In vivo: 60 mg/kg i.g. daily, 1 h after procedure, for 6 d In vitro: 100–800 ng/mL for 24 h	In vivo: ↓ ROS, 3-NT, and 8-OHdG ↑ GPx, CAT, SOD, and GSH/GSSG ratio In vitro: ↑ Cell viability ↓ ROS, ↑ GPx, CAT, SOD, and GSH/GSSG ratio ↓ TNF-α, IL-1β, and IL-6 ↓ Neuronal apoptosis ↓ Cleaved caspase-3 and Bax, ↑ Bcl-2 ↓ HMGB1 and RAGE	[177]
Mangiferin	5281647	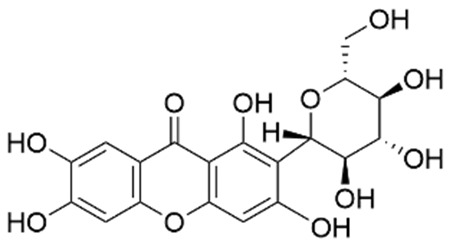	N/A	AGE-RAGE/MAPK pathway	LADO/R-induced myocardial IRI with STZ-induced diabetes in male albino Wistar rats	40 mg/kg i.p. daily, 28 d before procedure, for 28 d (sacrificed shortly after procedure)	↓ Cardiac oedema, inflammation, and necrosis ↓ Myofibres and mitochondria damage ↓ Blood glucose level, CK-MB, and LDH ↑ Haemodynamic parameters (SAP, MAP, DAP and HR) and ±LVdP/dt, ↓ LVEDP ↑ GSH, SOD, and CAT, ↓ MDA ↓ TNF-α and IL-6, NF-κB p65 ↓ Myocardial apoptosis ↑ Bcl-2, ↓ Bax and caspase-3 ↓ AGEs, RAGE, p38, and p-JNK, ↑ p-ERK1/2	[178]
Kaempferol	5280863	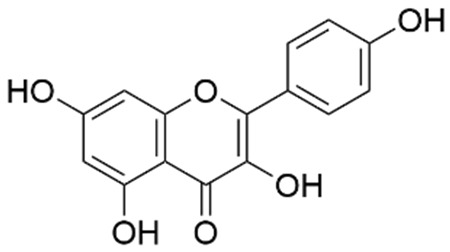	N/A	AGE-RAGE/MAPK pathway	LADO/R-induced myocardial IRI with STZ-induced diabetes in male albino Wistar rats	20 mg/kg i.p. daily, 4 d after STZ administration, for 28 d (sacrificed shortly after procedure)	↓ Cardiac oedema, inflammation, and necrosis ↓ Mitochondria damage ↓ Blood glucose level ↑ Haemodynamic parameters (SAP, MAP, DAP and HR) and ±LVdP/dt, ↓ LVEDP ↓ Serum TNF-α and IL-6 ↓ NF-κB p65 ↑ GSH, SOD, and CAT, ↓ MDA, CK-MB, and LDH ↓ Myocardial apoptosis ↑ Bcl-2, ↓ Bax and caspase-3 ↓ AGEs, RAGE, p38 and p-JNK, ↑ p-ERK1/2	[179]
Chrysin	5281607	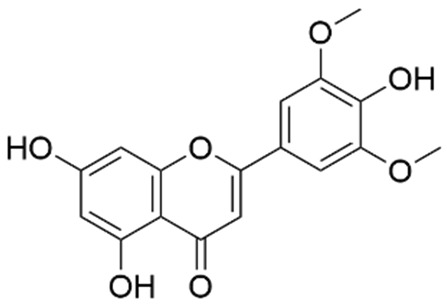	N/A	AGE-RAGE pathway	LADO/R-induced myocardial IRI with STZ-induced diabetes in male albino Wistar rats	60 mg/kg i.g. daily before procedure, for 28 d (sacrificed shortly after procedure)	↓ Cardiac oedema, inflammation, and necrosis ↓ Myofibres and mitochondria damage ↓ CK-MB, ↑ LDH ↑ MAP and ±LVdP/dtmax, ↓ LVEDP ↑ GSH and CAT, ↓ 8-OHdG, and TBARS ↓ NF-κB, IKK-β, TNF-α, MPO, and CRP ↓ Apoptosis ↑ PPAR-γ, Nrf2, and HO-1, ↓ RAGE	[180]
Artesunate	6917864	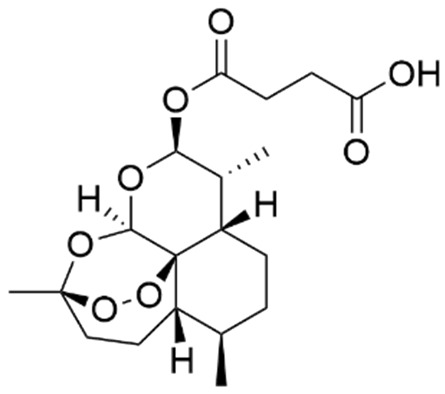	N/A	HMGB1-RAGE pathway	PTO/R-induced hepatic IRI in male albino Wistar rats	50 mg/kg, i.p. at the beginning of the reperfusion period (sacrificed shortly after procedure)	↓ Histopathological liver injury ↓ ALT, AST, and LDH ↓ ICAM-1, MPO, and MDA, ↑ GSH and SOD ↓ pS536-NF-κB p65, TNF-α, and IL-6 ↓ NLRP3, ASC, cleaved caspase-1, and caspase-11 ↓ GSDMD-NT, IL-18, and IL-1β ↑ Bcl-2, ↓ Bax ↓ HMGB1, RAGE, TLR4, MyD88 and TRAF6	[181]
Linalool	6549	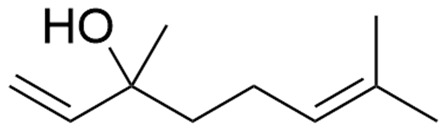	>TLR4/MD2 complex: −4.55 Kcal/mol with Lys109 and Arg69 >IKK: −4.80 Kcal/mol with Asp166 and Phe26 >Keap1/Nrf2: −5.61 Kcal/mol with ALA556, ALA510, and Gly509 >RAGE: −5.43 Kcal/mol with Arg66, Glu44, and Tyr341	HMGB1/TLR4/RAGE/NF-κB pathway Keap1/Nrf2/HO-1/NQO-1 pathway	PTO/R-induced hepatic IRI in male albino Wistar rats	100, 200 mg/kg/day i.g. daily before procedure, for 2 w (sacrificed shortly after procedure)	↓ Hepatic congestion, hepatocellular vacuolation and necrosis ↓ ALT, AST, ALP, and LDH ↓ MDA, NO, and ROS, ↑ SOD, CAT, GST, and GSH ↓ ICAM-1, MPO, TNF-α, IL-6, IL-1β and NF-κB, ↑ IL-10 ↓ caspase 3 and 9 ↓ Bax, ↑ Bcl2 ↓ HMGB1, TLR4, and RAGE ↑ Nrf-2, HO-1, and NQO-1	[182]
Luteolin	5280445	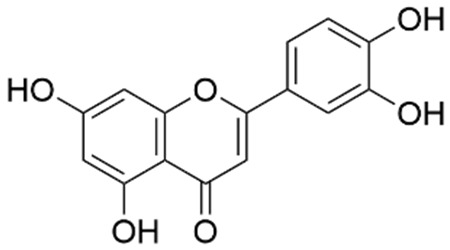	N/A	P2X7R–RAGE–TLR4 axis	In vivo: LPS/ATP co-stimulated sepsis in male C57BL/6 mice In vitro: peritoneal macrophages treated with LPS/ATP	In vivo: 30 and 60 mg/kg (sacrificed 48 h after procedure) In vitro: 0.32, 1.6 and 8 μM	In vivo: ↓ Liver histopathological injury ↓ ALT, AST and IL-1β ↓ HMGB1, NE, P2X7R, and MPO In vitro: ↓ Cytoplasmic aggregation of HMGB1 ↓ Caspase-1	[183]
Glycyrrhizin	14982	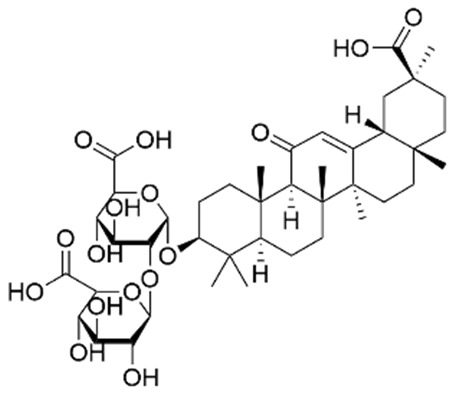	N/A	HMGB1-RAGE/TLR4-MAPK/NF-κB pathway	In vivo: CLP-induced sepsis in male Sprague-Dawley rats In vitro: Rat alveolar macrophage NR8383 cells treated with LPS or HMGB1	In vivo: 1 to 20 mg/kg i.v. daily, 12 h after procedure, for 3 d (sacrificed 0, 12, 30, and 60 h after procedure) In vitro: 10, 50 and 100 μg/mL	In vivo: ↑ Survival rate ↓ HMGB1, TNF-α, IL-1, HMGB1, and IL-6 ↓ Interactions between HMGB1 and RAGE or TLR4 In vitro: ↓ HMGB1, TNF-α, IL-1, and IL-6 ↓ p-IκBα, p-JNK, p-p38, and p-ERK	[184]
Papaverine	4680	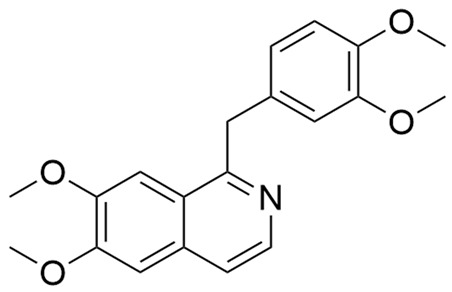	N/A	HMGB1-RAGE pathway	In vivo: CLP-induced sepsis in female ICR mice In vitro: RAW264.7 cells and BMM treated with HMGB1	In vivo: 20 mg/kg i.p. at 1 and 24 h after procedure In vitro: 3 and 30 μM	In vivo: ↑ Survival rate In vitro: ↓ IL-6 and TNF-α ↓ HMGB1/RAGE interaction	[185]
					FIP-induced sepsis in male Sprague-Dawley albino rats	20 and 40 mg/kg/d i.p.1 d after procedure, for 5 d (sacrificed at day 6)	↓ Axonal damage ↓ MDA and LA ↓ TNF-α, CRP, and IL-6 ↑ sRAGE, ↓ HMGB1	[186]
					FIP -induced sepsis in male Wistar rats	20 and 40 mg/kg/d i.p. 1 h after procedure (sacrificed 24 h after procedure)	↓ Lung histopathological injury ↓ HU value of lung ↑ PaO2 ↓ MDA and LA ↓ TNF-α and CRP ↑ sRAGE, ↓HMGB1	[187]
Berberine	2353	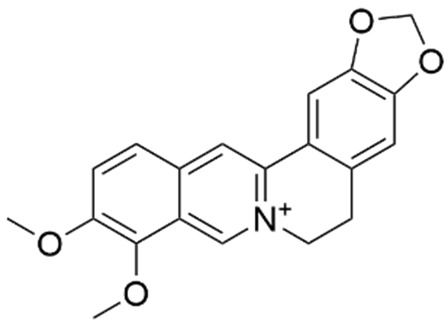	> HMGB1: −7.69 Kcal/mol	HMGB1-RAGE pathway	In vivo: CLP-induced sepsis in male C57BL/6 mice In vitro: primary mouse microglia and astrocytes treated with LPS or HMGB1	In vivo: 10 mg/kg 30 min before procedure In vitro: 5 μM	In vivo: ↑ Neo-neurons ↓ Cognitive impairment ↓ TNF-α, IL-1α, and C1qA In vitro: ↓ Microglia and astrocytes activation	[188]
Oxymatrine	114850	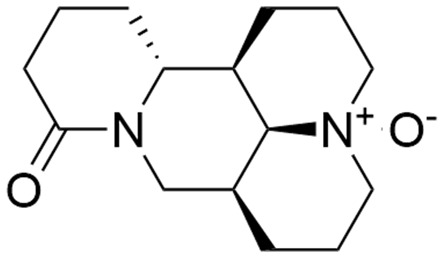	N/A	HMGB1/RAGE/NF-κB pathway	In vivo: CLP-induced sepsis in C57BL/6 J mice In vitro: THP-1 monocytes treated with LPS	In vivo: 80 mg/kg i.p. 6 h before procedure (sacrificed 12 h after procedure) In vitro: 40 and 80 μM	In vivo: ↑ Survival rate ↓ Lung histopathological injury ↓ Kidney histopathological injury ↓ IL-6, TNF-α, MCP-1 and VCAM-1 ↓ p-p65/p65 ratio, p-IκB-α, NF-kB p65 nuclear/cytosolic ratio, ↑ IκBα ↓ RAGE In vitro: ↓ IL-1β, IL-6, TNF-α, and MCP-1 ↓ p-p65/p65 ratio, p-IκBα, NF-kB p65 nuclear/cytosolic ratio, ↑ IκBα	[189]
Andrographolide	5318517	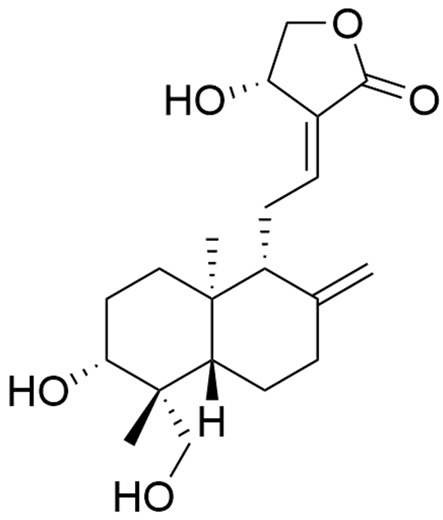	>RAGE: −7.5 kcal/mol with ASN-100, ASN-332, and MET-330 KD = 49.30 μM (SPR)	RAGE/PI3K/AKT/mTOR pathway	In vivo: CLP-induced sepsis in male C57BL/6 mice In vitro: MH-S alveolar macrophage line treated with LPS	In vivo: 20 mg/kg, i.p., q12 h before procedure for 3 d (sacrificed 24 h after procedure) In vitro: 10 µg/mL pretreated for 4 h	In vivo: ↑ Survival rate ↓ Lung histopathological injury ↓ IL-1β, IL-6, IL-18, and TNF-α, ↑ IL-10 ↓ NLRP3 and Caspase-1 ↑ LC3, LC3-II In vitro: ↓ IL-1β, IL-6, and TNF-α, ↑ IL-10 ↓ IL-18, NLRP3, Caspase-1 p20, and GSDMD-NT ↑ LC3--II ↓ p-PI3K, p-AKT, and p-mTOR	[190]
Anemoside B4	71307558	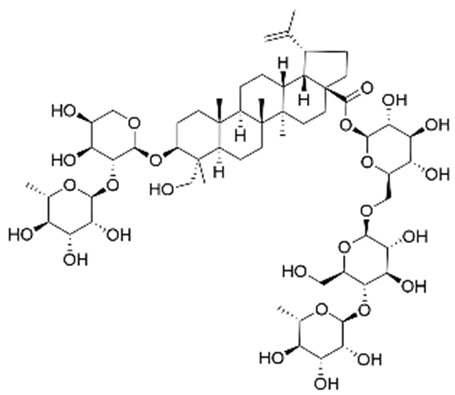	>RAGE: -7.152 kcal/mol with GLU-108, GLN-100, SER-65, GLY-69, VAL-63, LYS-62, and TRP-6 KD = 3.86 μM (SPR)	AGE-RAGE pathway	In vivo: CLP-induced sepsis in male C57BL/6 mice In vitro: RAW264.7 cells treated with LPS	In vivo: 50 and 100 mg/kg i.p. immediately post-surgery (sacrificed 24 h after procedure) In vitro: 0, 3, 6, 12, 24, 48 and 100 μM pretreated for 4 h	In vivo: ↓ Lung histopathological injury ↓ TNF-α, IL-6, and IL-1β ↓ MDA and DHE, ↑ GSH ↓ PTGS2 and RAGE, ↑ SLC7A11 and GPX4 ↓ HO-1, ↑ Nrf2 In vitro: ↑ Cell viability ↓ TNF-α, IL-1β, and IL-6 ↓ MDA, ↑ GSH ↓ PTGS2, ↑ GPX4 and SLC7A11	[191]

MCAO/R, middle cerebral artery occlusion/reperfusion; IRI, ischemia/reperfusion injury; OGD/R, oxygen-glucose deprivation/reperfusion; i.g., intragastric; N/A, not available; HMGB1, high mobility group box 1; RAGE, receptor for advanced glycation end-products; ROS, reactive oxygen species; 3-NT, 3-nitrotyrosine; 8-OHdG, 8-hydroxydeoxyguanosine; GPx, glutathione peroxidase; CAT, catalase; SOD, superoxide dismutase; GSH, glutathione; GSSG, glutathione disulphide; TNF-α, tumour necrosis factor-alpha; IL-1β, interleukin-1 beta; Bax, Bcl2 associated X; Bcl-2, B-cell lymphoma 2; MAPK, mitogen-activated protein kinase; LADO/R, left anterior descending artery occlusion/reperfusion; STZ, streptozotocin; i.p., intraperitoneal; CK-MB, creatine kinase-MB; LDH, lactate dehydrogenase; SAP, systolic arterial pressure; MAP, mean arterial pressure; DAP, diastolic arterial pressure; HR, heart rate; LVdP/dt, left ventricular pressure change over time; LVEDP, left ventricular end-diastolic pressure; MDA, malondialdehyde; NF-κB, nuclear factor-kappa B; AGEs, advanced glycation end-products; p-JNK, phosphorylated c-Jun N-terminal kinase; p-ERK1/2, phosphorylated extracellular signal-regulated kinase 1/2; 8-OHdG, 8-hydroxy-2’-deoxyguanosine; TBARS, thiobarbituric acid reactive substances; IKK-β, i-kappa-B kinase beta; MPO, myeloperoxidase; CRP, c-reactive protein; PPAR-γ, peroxisome proliferator-activated receptor gamma; Nrf2, nuclear factor erythroid 2-related factor 2; HO-1, heme oxygenase-1; PTO/R, portal triad occlusion/reperfusion; ALT, alanine transaminase; AST, aspartate aminotransferase; ICAM-1, intercellular adhesion molecule 1; NLRP3, NLR family pyrin domain containing 3; ASC, apoptosis-associated speck-like protein containing a CARD; GSDMD-NT, Gasdermin D, N-terminal; TLR4, Toll-like receptor 4; MyD88, myeloid differentiation primary response 88; TRAF6, TNF receptor-associated factor 6; MD2, myeloid differentiation factor 2; Keap1, kelch-like ECH-associated protein 1; ALP, alkaline phosphatase; NO, nitric Oxide; NQO-1, NAD(P)H quinone dehydrogenase 1; LPS, lipopolysaccharide; ATP, adenosine triphosphate; P2X7R, P2X purinoceptor 7; NE, neutrophil elastase; CLP, caecal ligation and puncture; DHE, Dihydroethidium; PI3K, phosphoinositide 3-kinase; mTOR, mechanistic target of rapamycin kinase; LC3, microtubule-associated protein 1A/1B-light chain 3; i.v., intravenous; IκBα, inhibitor of nuclear factor kappa B; MCP-1, monocyte chemoattractant protein-1; VCAM-1, vascular cell adhesion molecule-1; FIP, faecal-induced peritonitis; LA, lactic acid.

## Data Availability

No new data were created or analysed in this study. Data sharing is not applicable to this article.

## Abbreviations

The following abbreviations are used in this manuscript:

abRAGE	anti-RAGE antibodies
AGEs	advanced glycation end-products
AD	Alzheimer’s disease
ADAS-cog	Alzheimer’s Disease Assessment Scale cognitive portion
ADCS-ADL	Alzheimer’s Disease Cooperative Study–Activities of Daily Living
ALI/ARDS	acute lung injury or acute respiratory distress syndrome
ATP	adenosine triphosphate
Aβ	amyloid-β
BALF	bronchoalveolar lavage fluid
BBB	blood–brain barrier
CDR-sb	Clinical Dementia Rating Scale Sum of Boxes
CK-MB	creatine kinase-MB
CLP	caecal ligation and puncture
DAMPs	damage-associated molecular patterns
DNA	deoxyribonucleic acid
ERK	extracellular signal-regulated kinase
FIP	faecal-induced peritonitis
HMGB1	high mobility group box-1
HO-1	heme oxygenase-1
HSP70	heat shock protein 70
IL-6	interleukin-6
IRI	ischaemia–reperfusion injury
JAK	Janus kinase
Keap1	kelch-like ECH-associated protein 1
LPS	lipopolysaccharide
MAPK	mitogen-activated protein kinase
mDia1	mammalian diaphanous-related formin 1
MODS	multiple organ dysfunction syndrome
mTOR	mechanistic target of rapamycin kinase
MyD88	myeloid differentiation primary response 88
NF-κB	nuclear factor-kappa B
NLRP3	NLR family pyrin domain containing 3
NQO-1	NAD(P)H quinone dehydrogenase 1
Nrf2	nuclear factor erythroid 2-related factor 2
P2X7R	P2X purinoceptor 7
PAMPs	pathogen-associated molecular patterns
PI3K	phosphoinositide 3-kinase
PPAR-γ	peroxisome proliferator-activated receptor gamma
Rac1	ras-related C3 botulinum toxin substrate 1
RAGE	receptor for advanced glycation end-products
ROS	reactive oxygen species
sRAGE	soluble RAGE
STAT	signal transducer and activator of transcription
TLRs	Toll-like receptors
TNF-α	tumour necrosis factor alpha
TRAF6	tumour necrosis factor receptor-associated factor 6

## References

[1] Meyer N.J., Gattinoni L., Calfee C.S. (2021). Acute respiratory distress syndrome. Lancet.

[2] Iannuzzi J.P., King J.A., Leong J.H., Quan J., Windsor J.W., Tanyingoh D., Coward S., Forbes N., Heitman S.J., Shaheen A.A. (2022). Global Incidence of Acute Pancreatitis Is Increasing Over Time: A Systematic Review and Meta-Analysis. Gastroenterology.

[3] Collaborators G.G.S. (2025). Global, regional, and national sepsis incidence and mortality, 1990–2021: A systematic analysis. Lancet Glob. Health.

[4] Schepers N.J., Bakker O.J., Besselink M.G., Ahmed Ali U., Bollen T.L., Gooszen H.G., van Santvoort H.C., Bruno M.J. (2019). Impact of characteristics of organ failure and infected necrosis on mortality in necrotising pancreatitis. Gut.

[5] Shi N., Liu T., de la Iglesia-Garcia D., Deng L., Jin T., Lan L., Zhu P., Hu W., Zhou Z., Singh V. (2020). Duration of organ failure impacts mortality in acute pancreatitis. Gut.

[6] Machicado J.D., Gougol A., Tan X., Gao X., Paragomi P., Pothoulakis I., Talukdar R., Kochhar R., Goenka M.K., Gulla A. (2021). Mortality in acute pancreatitis with persistent organ failure is determined by the number, type, and sequence of organ systems affected. United Eur. Gastroenterol. J..

[7] Eltzschig H.K., Eckle T. (2011). Ischemia and reperfusion--from mechanism to translation. Nat. Med..

[8] Stoll G., Nieswandt B. (2019). Thrombo-inflammation in acute ischaemic stroke-implications for treatment. Nat. Rev. Neurol..

[9] Heusch G. (2020). Myocardial ischaemia-reperfusion injury and cardioprotection in perspective. Nat. Rev. Cardiol..

[10] Hirao H., Nakamura K., Kupiec-Weglinski J.W. (2022). Liver ischaemia-reperfusion injury: A new understanding of the role of innate immunity. Nat. Rev. Gastroenterol. Hepatol..

[11] Bayır H., Dixon S.J., Tyurina Y.Y., Kellum J.A., Kagan V.E. (2023). Ferroptotic mechanisms and therapeutic targeting of iron metabolism and lipid peroxidation in the kidney. Nat. Rev. Nephrol..

[12] Fleischmann C., Scherag A., Adhikari N.K., Hartog C.S., Tsaganos T., Schlattmann P., Angus D.C., Reinhart K. (2016). Assessment of Global Incidence and Mortality of Hospital-treated Sepsis. Current Estimates and Limitations. Am. J. Respir. Crit. Care Med..

[13] Hudson B.I., Lippman M.E. (2018). Targeting RAGE Signaling in Inflammatory Disease. Annu. Rev. Med..

[14] Furman D., Campisi J., Verdin E., Carrera-Bastos P., Targ S., Franceschi C., Ferrucci L., Gilroy D.W., Fasano A., Miller G.W. (2019). Chronic inflammation in the etiology of disease across the life span. Nat. Med..

[15] Zindel J., Kubes P. (2020). DAMPs, PAMPs, and LAMPs in Immunity and Sterile Inflammation. Annu. Rev. Pathol..

[16] Huang Y., Jiang W., Zhou R. (2024). DAMP sensing and sterile inflammation: Intracellular, intercellular and inter-organ pathways. Nat. Rev. Immunol..

[17] Janeway C.A., Medzhitov R. (2002). Innate immune recognition. Annu. Rev. Immunol..

[18] Linkermann A., Stockwell B.R., Krautwald S., Anders H.J. (2014). Regulated cell death and inflammation: An auto-amplification loop causes organ failure. Nat. Rev. Immunol..

[19] Gong T., Liu L., Jiang W., Zhou R. (2020). DAMP-sensing receptors in sterile inflammation and inflammatory diseases. Nat. Rev. Immunol..

[20] Prantner D., Nallar S., Vogel S.N. (2020). The role of RAGE in host pathology and crosstalk between RAGE and TLR4 in innate immune signal transduction pathways. FASEB J..

[21] Kim H.J., Jeong M.S., Jang S.B. (2021). Molecular Characteristics of RAGE and Advances in Small-Molecule Inhibitors. Int. J. Mol. Sci..

[22] Constien R., Forde A., Liliensiek B., Gröne H.J., Nawroth P., Hämmerling G., Arnold B. (2001). Characterization of a novel EGFP reporter mouse to monitor Cre recombination as demonstrated by a Tie2 Cre mouse line. Genesis.

[23] Atanasov A.G., Zotchev S.B., Dirsch V.M., Supuran C.T. (2021). Natural products in drug discovery: Advances and opportunities. Nat. Rev. Drug Discov..

[24] Schmidt A.M., Vianna M., Gerlach M., Brett J., Ryan J., Kao J., Esposito C., Hegarty H., Hurley W., Clauss M. (1992). Isolation and characterization of two binding proteins for advanced glycosylation end products from bovine lung which are present on the endothelial cell surface. J. Biol. Chem..

[25] Neeper M., Schmidt A.M., Brett J., Yan S.D., Wang F., Pan Y.C., Elliston K., Stern D., Shaw A. (1992). Cloning and expression of a cell surface receptor for advanced glycosylation end products of proteins. J. Biol. Chem..

[26] Malherbe P., Richards J.G., Gaillard H., Thompson A., Diener C., Schuler A., Huber G. (1999). cDNA cloning of a novel secreted isoform of the human receptor for advanced glycation end products and characterization of cells co-expressing cell-surface scavenger receptors and Swedish mutant amyloid precursor protein. Brain Res. Mol. Brain Res..

[27] Xie J., Reverdatto S., Frolov A., Hoffmann R., Burz D.S., Shekhtman A. (2008). Structural basis for pattern recognition by the receptor for advanced glycation end products (RAGE). J. Biol. Chem..

[28] Bongarzone S., Savickas V., Luzi F., Gee A.D. (2017). Targeting the Receptor for Advanced Glycation Endproducts (RAGE): A Medicinal Chemistry Perspective. J. Med. Chem..

[29] Fritz G. (2011). RAGE: A single receptor fits multiple ligands. Trends Biochem. Sci..

[30] Yatime L., Andersen G.R. (2013). Structural insights into the oligomerization mode of the human receptor for advanced glycation end-products. FEBS J..

[31] Koch M., Chitayat S., Dattilo B.M., Schiefner A., Diez J., Chazin W.J., Fritz G. (2010). Structural basis for ligand recognition and activation of RAGE. Structure.

[32] Sturchler E., Galichet A., Weibel M., Leclerc E., Heizmann C.W. (2008). Site-specific blockade of RAGE-Vd prevents amyloid-beta oligomer neurotoxicity. J. Neurosci..

[33] Kierdorf K., Fritz G. (2013). RAGE regulation and signaling in inflammation and beyond. J. Leukoc. Biol..

[34] Ostendorp T., Leclerc E., Galichet A., Koch M., Demling N., Weigle B., Heizmann C.W., Kroneck P.M., Fritz G. (2007). Structural and functional insights into RAGE activation by multimeric S100B. Embo J..

[35] Katsuoka F., Kawakami Y., Arai T., Imuta H., Fujiwara M., Kanma H., Yamashita K. (1997). Type II alveolar epithelial cells in lung express receptor for advanced glycation end products (RAGE) gene. Biochem. Biophys. Res. Commun..

[36] Fehrenbach H., Kasper M., Tschernig T., Shearman M.S., Schuh D., Müller M. (1998). Receptor for advanced glycation endproducts (RAGE) exhibits highly differential cellular and subcellular localisation in rat and human lung. Cell. Mol. Biol. (Noisy-Le-Grand).

[37] Chen Y., Yan S.S., Colgan J., Zhang H.P., Luban J., Schmidt A.M., Stern D., Herold K.C. (2004). Blockade of late stages of autoimmune diabetes by inhibition of the receptor for advanced glycation end products. J. Immunol..

[38] Bierhaus A., Stern D.M., Nawroth P.P. (2006). RAGE in inflammation: A new therapeutic target?. Curr. Opin. Investig. Drugs.

[39] Moser B., Desai D.D., Downie M.P., Chen Y., Yan S.F., Herold K., Schmidt A.M., Clynes R. (2007). Receptor for advanced glycation end products expression on T cells contributes to antigen-specific cellular expansion in vivo. J. Immunol..

[40] Brett J., Schmidt A.M., Yan S.D., Zou Y.S., Weidman E., Pinsky D., Nowygrod R., Neeper M., Przysiecki C., Shaw A. (1993). Survey of the distribution of a newly characterized receptor for advanced glycation end products in tissues. Am. J. Pathol..

[41] Schmidt A.M., Yan S.D., Brett J., Mora R., Nowygrod R., Stern D. (1993). Regulation of human mononuclear phagocyte migration by cell surface-binding proteins for advanced glycation end products. J. Clin. Investig..

[42] Wendt T.M., Tanji N., Guo J., Kislinger T.R., Qu W., Lu Y., Bucciarelli L.G., Rong L.L., Moser B., Markowitz G.S. (2003). RAGE drives the development of glomerulosclerosis and implicates podocyte activation in the pathogenesis of diabetic nephropathy. Am. J. Pathol..

[43] Demling N., Ehrhardt C., Kasper M., Laue M., Knels L., Rieber E.P. (2006). Promotion of cell adherence and spreading: A novel function of RAGE, the highly selective differentiation marker of human alveolar epithelial type I cells. Cell Tissue Res..

[44] Harja E., Bu D.X., Hudson B.I., Chang J.S., Shen X., Hallam K., Kalea A.Z., Lu Y., Rosario R.H., Oruganti S. (2008). Vascular and inflammatory stresses mediate atherosclerosis via RAGE and its ligands in apoE-/- mice. J. Clin. Investig..

[45] Li J., Schmidt A.M. (1997). Characterization and functional analysis of the promoter of RAGE, the receptor for advanced glycation end products. J. Biol. Chem..

[46] Li J., Qu X., Schmidt A.M. (1998). Sp1-binding elements in the promoter of RAGE are essential for amphoterin-mediated gene expression in cultured neuroblastoma cells. J. Biol. Chem..

[47] Hudson B.I., Kalea A.Z., Del Mar Arriero M., Harja E., Boulanger E., D’Agati V., Schmidt A.M. (2008). Interaction of the RAGE cytoplasmic domain with diaphanous-1 is required for ligand-stimulated cellular migration through activation of Rac1 and Cdc42. J. Biol. Chem..

[48] Deane R., Singh I., Sagare A.P., Bell R.D., Ross N.T., LaRue B., Love R., Perry S., Paquette N., Deane R.J. (2012). A multimodal RAGE-specific inhibitor reduces amyloid β-mediated brain disorder in a mouse model of Alzheimer disease. J. Clin. Investig..

[49] Jules J., Maiguel D., Hudson B.I. (2013). Alternative splicing of the RAGE cytoplasmic domain regulates cell signaling and function. PLoS ONE.

[50] Hong Y., Shen C., Yin Q., Sun M., Ma Y., Liu X. (2016). Effects of RAGE-Specific Inhibitor FPS-ZM1 on Amyloid-β Metabolism and AGEs-Induced Inflammation and Oxidative Stress in Rat Hippocampus. Neurochem. Res..

[51] Huang J.S., Guh J.Y., Chen H.C., Hung W.C., Lai Y.H., Chuang L.Y. (2001). Role of receptor for advanced glycation end-product (RAGE) and the JAK/STAT-signaling pathway in AGE-induced collagen production in NRK-49F cells. J. Cell Biochem..

[52] Kaji Y., Usui T., Ishida S., Yamashiro K., Moore T.C., Moore J., Yamamoto Y., Yamamoto H., Adamis A.P. (2007). Inhibition of diabetic leukostasis and blood-retinal barrier breakdown with a soluble form of a receptor for advanced glycation end products. Investig. Ophthalmol. Vis. Sci..

[53] Reynolds P.R., Kasteler S.D., Cosio M.G., Sturrock A., Huecksteadt T., Hoidal J.R. (2008). RAGE: Developmental expression and positive feedback regulation by Egr-1 during cigarette smoke exposure in pulmonary epithelial cells. Am. J. Physiol. Lung Cell Mol. Physiol..

[54] Takeuchi A., Yamamoto Y., Munesue S., Harashima A., Watanabe T., Yonekura H., Yamamoto H., Tsuchiya H. (2013). Low molecular weight heparin suppresses receptor for advanced glycation end products-mediated expression of malignant phenotype in human fibrosarcoma cells. Cancer Sci..

[55] Li Y., Wu R., Tian Y., Yu M., Tang Y., Cheng H., Tian Z. (2015). RAGE/NF-κB signaling mediates lipopolysaccharide induced acute lung injury in neonate rat model. Int. J. Clin. Exp. Med..

[56] Schmidt A.M., Yan S.D., Yan S.F., Stern D.M. (2000). The biology of the receptor for advanced glycation end products and its ligands. Biochim. Biophys. Acta.

[57] Kosaka T., Fukui R., Matsui M., Kurosaka Y., Nishimura H., Tanabe M., Takakura Y., Iwai K., Waki T., Fujita T. (2014). RAGE, receptor of advanced glycation endoproducts, negatively regulates chondrocytes differentiation. PLoS ONE.

[58] Yonekura H., Yamamoto Y., Sakurai S., Petrova R.G., Abedin M.J., Li H., Yasui K., Takeuchi M., Makita Z., Takasawa S. (2003). Novel splice variants of the receptor for advanced glycation end-products expressed in human vascular endothelial cells and pericytes, and their putative roles in diabetes-induced vascular injury. Biochem. J..

[59] Koyama H., Yamamoto H., Nishizawa Y. (2007). RAGE and soluble RAGE: Potential therapeutic targets for cardiovascular diseases. Mol. Med..

[60] Emanuele E., D’Angelo A., Tomaino C., Binetti G., Ghidoni R., Politi P., Bernardi L., Maletta R., Bruni A.C., Geroldi D. (2005). Circulating levels of soluble receptor for advanced glycation end products in Alzheimer disease and vascular dementia. Arch. Neurol..

[61] Falcone C., Emanuele E., D’Angelo A., Buzzi M.P., Belvito C., Cuccia M., Geroldi D. (2005). Plasma levels of soluble receptor for advanced glycation end products and coronary artery disease in nondiabetic men. Arterioscler. Thromb. Vasc. Biol..

[62] Kalea A.Z., Schmidt A.M., Hudson B.I. (2009). RAGE: A novel biological and genetic marker for vascular disease. Clin. Sci..

[63] Scavello F., Zeni F., Milano G., Macrì F., Castiglione S., Zuccolo E., Scopece A., Pezone G., Tedesco C.C., Nigro P. (2021). Soluble Receptor for Advanced Glycation End-products regulates age-associated Cardiac Fibrosis. Int. J. Biol. Sci..

[64] Yamagishi S., Adachi H., Nakamura K., Matsui T., Jinnouchi Y., Takenaka K., Takeuchi M., Enomoto M., Furuki K., Hino A. (2006). Positive association between serum levels of advanced glycation end products and the soluble form of receptor for advanced glycation end products in nondiabetic subjects. Metabolism.

[65] Geroldi D., Falcone C., Emanuele E. (2006). Soluble receptor for advanced glycation end products: From disease marker to potential therapeutic target. Curr. Med. Chem..

[66] Cho H.J., Son S.M., Jin S.M., Hong H.S., Shin D.H., Kim S.J., Huh K., Mook-Jung I. (2009). RAGE regulates BACE1 and Abeta generation via NFAT1 activation in Alzheimer’s disease animal model. FASEB J..

[67] Zhang L., Postina R., Wang Y. (2009). Ectodomain shedding of the receptor for advanced glycation end products: A novel therapeutic target for Alzheimer’s disease. Cell Mol. Life Sci..

[68] Sims G.P., Rowe D.C., Rietdijk S.T., Herbst R., Coyle A.J. (2010). HMGB1 and RAGE in inflammation and cancer. Annu. Rev. Immunol..

[69] Yamamoto Y., Harashima A., Saito H., Tsuneyama K., Munesue S., Motoyoshi S., Han D., Watanabe T., Asano M., Takasawa S. (2011). Septic shock is associated with receptor for advanced glycation end products ligation of LPS. J. Immunol..

[70] Hori O., Brett J., Slattery T., Cao R., Zhang J., Chen J.X., Nagashima M., Lundh E.R., Vijay S., Nitecki D. (1995). The receptor for advanced glycation end products (RAGE) is a cellular binding site for amphoterin. Mediation of neurite outgrowth and co-expression of rage and amphoterin in the developing nervous system. J. Biol. Chem..

[71] Jangde N., Ray R., Rai V. (2020). RAGE and its ligands: From pathogenesis to therapeutics. Crit. Rev. Biochem. Mol. Biol..

[72] Dong H., Zhang Y., Huang Y., Deng H. (2022). Pathophysiology of RAGE in inflammatory diseases. Front. Immunol..

[73] Xie J., Burz D.S., He W., Bronstein I.B., Lednev I., Shekhtman A. (2007). Hexameric calgranulin C (S100A12) binds to the receptor for advanced glycated end products (RAGE) using symmetric hydrophobic target-binding patches. J. Biol. Chem..

[74] Dattilo B.M., Fritz G., Leclerc E., Kooi C.W., Heizmann C.W., Chazin W.J. (2007). The extracellular region of the receptor for advanced glycation end products is composed of two independent structural units. Biochemistry.

[75] Leclerc E., Fritz G., Weibel M., Heizmann C.W., Galichet A. (2007). S100B and S100A6 differentially modulate cell survival by interacting with distinct RAGE (receptor for advanced glycation end products) immunoglobulin domains. J. Biol. Chem..

[76] Verweij C.L. (2002). How RAGE turns in rage. Genes. Immun..

[77] Sirois C.M., Jin T., Miller A.L., Bertheloot D., Nakamura H., Horvath G.L., Mian A., Jiang J., Schrum J., Bossaller L. (2013). RAGE is a nucleic acid receptor that promotes inflammatory responses to DNA. J. Exp. Med..

[78] Ma W., Rai V., Hudson B.I., Song F., Schmidt A.M., Barile G.R. (2012). RAGE binds C1q and enhances C1q-mediated phagocytosis. Cell Immunol..

[79] Grunwald M.S., Ligabue-Braun R., Souza C.S., Heimfarth L., Verli H., Gelain D.P., Moreira J.C. (2017). Putative model for heat shock protein 70 complexation with receptor of advanced glycation end products through fluorescence proximity assays and normal mode analyses. Cell Stress. Chaperones.

[80] Somensi N., Brum P.O., de Miranda Ramos V., Gasparotto J., Zanotto-Filho A., Rostirolla D.C., da Silva Morrone M., Moreira J.C.F., Pens Gelain D. (2017). Extracellular HSP70 Activates ERK1/2, NF-kB and Pro-Inflammatory Gene Transcription Through Binding with RAGE in A549 Human Lung Cancer Cells. Cell Physiol. Biochem..

[81] Kay A.M., Simpson C.L., Stewart J.A. (2016). The Role of AGE/RAGE Signaling in Diabetes-Mediated Vascular Calcification. J. Diabetes Res..

[82] He M., Kubo H., Morimoto K., Fujino N., Suzuki T., Takahasi T., Yamada M., Yamaya M., Maekawa T., Yamamoto Y. (2011). Receptor for advanced glycation end products binds to phosphatidylserine and assists in the clearance of apoptotic cells. EMBO Rep..

[83] Friggeri A., Banerjee S., Biswas S., de Freitas A., Liu G., Bierhaus A., Abraham E. (2011). Participation of the receptor for advanced glycation end products in efferocytosis. J. Immunol..

[84] Chavakis T., Bierhaus A., Al-Fakhri N., Schneider D., Witte S., Linn T., Nagashima M., Morser J., Arnold B., Preissner K.T. (2003). The pattern recognition receptor (RAGE) is a counterreceptor for leukocyte integrins: A novel pathway for inflammatory cell recruitment. J. Exp. Med..

[85] Frommhold D., Kamphues A., Hepper I., Pruenster M., Lukic I.K., Socher I., Zablotskaya V., Buschmann K., Lange-Sperandio B., Schymeinsky J. (2010). RAGE and ICAM-1 cooperate in mediating leukocyte recruitment during acute inflammation in vivo. Blood.

[86] Wang L., Wu J., Guo X., Huang X., Huang Q. (2017). RAGE Plays a Role in LPS-Induced NF-κB Activation and Endothelial Hyperpermeability. Sensors.

[87] Scaffidi P., Misteli T., Bianchi M.E. (2002). Release of chromatin protein HMGB1 by necrotic cells triggers inflammation. Nature.

[88] Lotze M.T., Tracey K.J. (2005). High-mobility group box 1 protein (HMGB1): Nuclear weapon in the immune arsenal. Nat. Rev. Immunol..

[89] Kuniyasu H., Chihara Y., Takahashi T. (2003). Co-expression of receptor for advanced glycation end products and the ligand amphoterin associates closely with metastasis of colorectal cancer. Oncol. Rep..

[90] Orlova V.V., Choi E.Y., Xie C., Chavakis E., Bierhaus A., Ihanus E., Ballantyne C.M., Gahmberg C.G., Bianchi M.E., Nawroth P.P. (2007). A novel pathway of HMGB1-mediated inflammatory cell recruitment that requires Mac-1-integrin. EMBO J..

[91] LeBlanc P.M., Doggett T.A., Choi J., Hancock M.A., Durocher Y., Frank F., Nagar B., Ferguson T.A., Saleh M. (2014). An immunogenic peptide in the A-box of HMGB1 protein reverses apoptosis-induced tolerance through RAGE receptor. J. Biol. Chem..

[92] Xu J., Jiang Y., Wang J., Shi X., Liu Q., Liu Z., Li Y., Scott M.J., Xiao G., Li S. (2014). Macrophage endocytosis of high-mobility group box 1 triggers pyroptosis. Cell Death Differ..

[93] Yang J., Zhao Y., Zhang P., Li Y., Yang Y., Yang Y., Zhu J., Song X., Jiang G., Fan J. (2016). Hemorrhagic shock primes for lung vascular endothelial cell pyroptosis: Role in pulmonary inflammation following LPS. Cell Death Dis..

[94] Sarkar S. (2024). Pathological role of RAGE underlying progression of various diseases: Its potential as biomarker and therapeutic target. Naunyn Schmiedeberg’s Arch. Pharmacol..

[95] Wang H., Liao H., Ochani M., Justiniani M., Lin X., Yang L., Al-Abed Y., Wang H., Metz C., Miller E.J. (2004). Cholinergic agonists inhibit HMGB1 release and improve survival in experimental sepsis. Nat. Med..

[96] Yang H., Ochani M., Li J., Qiang X., Tanovic M., Harris H.E., Susarla S.M., Ulloa L., Wang H., DiRaimo R. (2004). Reversing established sepsis with antagonists of endogenous high-mobility group box 1. Proc. Natl. Acad. Sci. USA.

[97] Suda K., Kitagawa Y., Ozawa S., Saikawa Y., Ueda M., Ebina M., Yamada S., Hashimoto S., Fukata S., Abraham E. (2006). Anti-high-mobility group box chromosomal protein 1 antibodies improve survival of rats with sepsis. World J. Surg..

[98] van Zoelen M.A., Achouiti A., van der Poll T. (2011). The role of receptor for advanced glycation endproducts (RAGE) in infection. Crit. Care.

[99] Hofmann M.A., Drury S., Fu C., Qu W., Taguchi A., Lu Y., Avila C., Kambham N., Bierhaus A., Nawroth P. (1999). RAGE mediates a novel proinflammatory axis: A central cell surface receptor for S100/calgranulin polypeptides. Cell.

[100] Weigand M.A., Volkmann M., Schmidt H., Martin E., Böhrer H., Bardenheuer H.J. (2000). Neuron-specific enolase as a marker of fatal outcome in patients with severe sepsis or septic shock. Anesthesiology.

[101] Donato R. (2001). S100: A multigenic family of calcium-modulated proteins of the EF-hand type with intracellular and extracellular functional roles. Int. J. Biochem. Cell Biol..

[102] Wittkowski H., Sturrock A., van Zoelen M.A., Viemann D., van der Poll T., Hoidal J.R., Roth J., Foell D. (2007). Neutrophil-derived S100A12 in acute lung injury and respiratory distress syndrome. Crit. Care Med..

[103] Zhong X., Xie F., Chen L., Liu Z., Wang Q. (2020). S100A8 and S100A9 promote endothelial cell activation through the RAGE-mediated mammalian target of rapamycin complex 2 pathway. Mol. Med. Rep..

[104] Kiryushko D., Novitskaya V., Soroka V., Klingelhofer J., Lukanidin E., Berezin V., Bock E. (2006). Molecular mechanisms of Ca(2+) signaling in neurons induced by the S100A4 protein. Mol. Cell Biol..

[105] Park H., Adsit F.G., Boyington J.C. (2010). The 1.5 Å crystal structure of human receptor for advanced glycation endproducts (RAGE) ectodomains reveals unique features determining ligand binding. J. Biol. Chem..

[106] Xue J., Manigrasso M., Scalabrin M., Rai V., Reverdatto S., Burz D.S., Fabris D., Schmidt A.M., Shekhtman A. (2016). Change in the Molecular Dimension of a RAGE-Ligand Complex Triggers RAGE Signaling. Structure.

[107] Björk P., Björk A., Vogl T., Stenström M., Liberg D., Olsson A., Roth J., Ivars F., Leanderson T. (2009). Identification of human S100A9 as a novel target for treatment of autoimmune disease via binding to quinoline-3-carboxamides. PLoS Biol..

[108] Suresh R., Mosser D.M. (2013). Pattern recognition receptors in innate immunity, host defense, and immunopathology. Adv. Physiol. Educ..

[109] Liu L., Yang M., Kang R., Dai Y., Yu Y., Gao F., Wang H., Sun X., Li X., Li J. (2014). HMGB1-DNA complex-induced autophagy limits AIM2 inflammasome activation through RAGE. Biochem. Biophys. Res. Commun..

[110] Ruan B.H., Li X., Winkler A.R., Cunningham K.M., Kuai J., Greco R.M., Nocka K.H., Fitz L.J., Wright J.F., Pittman D.D. (2010). Complement C3a, CpG oligos, and DNA/C3a complex stimulate IFN-α production in a receptor for advanced glycation end product-dependent manner. J. Immunol..

[111] Teh B.K., Yeo J.G., Chern L.M., Lu J. (2011). C1q regulation of dendritic cell development from monocytes with distinct cytokine production and T cell stimulation. Mol. Immunol..

[112] Ishihara K., Tsutsumi K., Kawane S., Nakajima M., Kasaoka T. (2003). The receptor for advanced glycation end-products (RAGE) directly binds to ERK by a D-domain-like docking site. FEBS Lett..

[113] Kellow N.J., Coughlan M.T. (2015). Effect of diet-derived advanced glycation end products on inflammation. Nutr. Rev..

[114] Senatus L.M., Schmidt A.M. (2017). The AGE-RAGE Axis: Implications for Age-Associated Arterial Diseases. Front. Genet..

[115] Lander H.M., Tauras J.M., Ogiste J.S., Hori O., Moss R.A., Schmidt A.M. (1997). Activation of the receptor for advanced glycation end products triggers a p21(ras)-dependent mitogen-activated protein kinase pathway regulated by oxidant stress. J. Biol. Chem..

[116] Wautier M.P., Chappey O., Corda S., Stern D.M., Schmidt A.M., Wautier J.L. (2001). Activation of NADPH oxidase by AGE links oxidant stress to altered gene expression via RAGE. Am. J. Physiol. Endocrinol. Metab..

[117] Thornalley P.J., Battah S., Ahmed N., Karachalias N., Agalou S., Babaei-Jadidi R., Dawnay A. (2003). Quantitative screening of advanced glycation endproducts in cellular and extracellular proteins by tandem mass spectrometry. Biochem. J..

[118] Salehi M., Amiri S., Ilghari D., Hasham L.F.A., Piri H. (2023). The Remarkable Roles of the Receptor for Advanced Glycation End Products (RAGE) and Its Soluble Isoforms in COVID-19: The Importance of RAGE Pathway in the Lung Injuries. Indian. J. Clin. Biochem..

[119] Thornalley P.J. (1998). Cell activation by glycated proteins. AGE receptors, receptor recognition factors and functional classification of AGEs. Cell. Mol. Biol. (Noisy-Le-Grand).

[120] Zhang F., Su X., Huang G., Xin X.F., Cao E.H., Shi Y., Song Y. (2017). sRAGE alleviates neutrophilic asthma by blocking HMGB1/RAGE signalling in airway dendritic cells. Sci. Rep..

[121] Steenbeke M., Speeckaert R., Desmedt S., Glorieux G., Delanghe J.R., Speeckaert M.M. (2022). The Role of Advanced Glycation End Products and Its Soluble Receptor in Kidney Diseases. Int. J. Mol. Sci..

[122] Liu Y., Liang C., Liu X., Liao B., Pan X., Ren Y., Fan M., Li M., He Z., Wu J. (2010). AGEs increased migration and inflammatory responses of adventitial fibroblasts via RAGE, MAPK and NF-kappaB pathways. Atherosclerosis.

[123] Glade M.J., Smith K. (2015). Phosphatidylserine and the human brain. Nutrition.

[124] Creagh-Brown B.C., Quinlan G.J., Evans T.W., Burke-Gaffney A. (2010). The RAGE axis in systemic inflammation, acute lung injury and myocardial dysfunction: An important therapeutic target?. Intensive Care Med..

[125] Zhou M., Zhang Y., Shi L., Li L., Zhang D., Gong Z., Wu Q. (2024). Activation and modulation of the AGEs-RAGE axis: Implications for inflammatory pathologies and therapeutic interventions—A review. Pharmacol. Res..

[126] Cohen M.J., Carles M., Brohi K., Calfee C.S., Rahn P., Call M.S., Chesebro B.B., West M.A., Pittet J.F. (2010). Early release of soluble receptor for advanced glycation endproducts after severe trauma in humans. J. Trauma.

[127] Asgeirsson T., Zhang S., Khoo S.K., Resau J.H., Dujovny N., Senagore A.J. (2011). Serum adiponectin, resistin, and circulating soluble receptor for advanced glycation end products in colectomy patients. Mediat. Inflamm..

[128] Uchida T., Shirasawa M., Ware L.B., Kojima K., Hata Y., Makita K., Mednick G., Matthay Z.A., Matthay M.A. (2006). Receptor for advanced glycation end-products is a marker of type I cell injury in acute lung injury. Am. J. Respir. Crit. Care Med..

[129] Zhang H., Tasaka S., Shiraishi Y., Fukunaga K., Yamada W., Seki H., Ogawa Y., Miyamoto K., Nakano Y., Hasegawa N. (2008). Role of soluble receptor for advanced glycation end products on endotoxin-induced lung injury. Am. J. Respir. Crit. Care Med..

[130] Kang R., Chen R., Xie M., Cao L., Lotze M.T., Tang D., Zeh H.J. (2016). The Receptor for Advanced Glycation End Products Activates the AIM2 Inflammasome in Acute Pancreatitis. J. Immunol..

[131] Deng M., Tang Y., Li W., Wang X., Zhang R., Zhang X., Zhao X., Liu J., Tang C., Liu Z. (2018). The Endotoxin Delivery Protein HMGB1 Mediates Caspase-11-Dependent Lethality in Sepsis. Immunity.

[132] Wang H., Wang T., Yuan Z., Cao Y., Zhou Y., He J., Shen Y., Zeng N., Dai L., Wen F. (2018). Role of Receptor for Advanced Glycation End Products in Regulating Lung Fluid Balance in Lipopolysaccharide-induced Acute Lung Injury and Infection-Related Acute Respiratory Distress Syndrome. Shock.

[133] Ding X., Jin S., Tian W., Zhang Y., Xu L., Zhang T., Chen Z., Niu F., Li Q. (2025). Role of Caspase-1/Caspase-11-HMGB1-RAGE/TLR4 Signaling in the Exacerbation of Extrapulmonary Sepsis-Induced Lung Injury by Mechanical Ventilation. Shock.

[134] Calfee C.S., Ware L.B., Eisner M.D., Parsons P.E., Thompson B.T., Wickersham N., Matthay M.A. (2008). Plasma receptor for advanced glycation end products and clinical outcomes in acute lung injury. Thorax.

[135] Mauri T., Masson S., Pradella A., Bellani G., Coppadoro A., Bombino M., Valentino S., Patroniti N., Mantovani A., Pesenti A. (2010). Elevated plasma and alveolar levels of soluble receptor for advanced glycation endproducts are associated with severity of lung dysfunction in ARDS patients. Tohoku J. Exp. Med..

[136] Jabaudon M., Futier E., Roszyk L., Chalus E., Guerin R., Petit A., Mrozek S., Perbet S., Cayot-Constantin S., Chartier C. (2011). Soluble form of the receptor for advanced glycation end products is a marker of acute lung injury but not of severe sepsis in critically ill patients. Crit. Care Med..

[137] Jabaudon M., Blondonnet R., Roszyk L., Bouvier D., Audard J., Clairefond G., Fournier M., Marceau G., Déchelotte P., Pereira B. (2015). Soluble Receptor for Advanced Glycation End-Products Predicts Impaired Alveolar Fluid Clearance in Acute Respiratory Distress Syndrome. Am. J. Respir. Crit. Care Med..

[138] Jabaudon M., Berthelin P., Pranal T., Roszyk L., Godet T., Faure J.S., Chabanne R., Eisenmann N., Lautrette A., Belville C. (2018). Receptor for advanced glycation end-products and ARDS prediction: A multicentre observational study. Sci. Rep..

[139] Edriss H., Molehin A.J., Gavidia R., Selvan K., Nugent K. (2020). Association between acute respiratory failure requiring mechanical ventilation and the production of advanced glycation end products. J. Investig. Med..

[140] Jabaudon M., Pereira B., Laroche E., Roszyk L., Blondonnet R., Audard J., Godet T., Futier E., Bazin J.E., Sapin V. (2021). Changes in Plasma Soluble Receptor for Advanced Glycation End-Products Are Associated with Survival in Patients with Acute Respiratory Distress Syndrome. J. Clin. Med..

[141] Lindström O., Tukiainen E., Kylänpää L., Mentula P., Rouhiainen A., Puolakkainen P., Rauvala H., Repo H. (2009). Circulating levels of a soluble form of receptor for advanced glycation end products and high-mobility group box chromosomal protein 1 in patients with acute pancreatitis. Pancreas.

[142] Kocsis A.K., Szabolcs A., Hofner P., Takács T., Farkas G., Boda K., Mándi Y. (2009). Plasma concentrations of high-mobility group box protein 1, soluble receptor for advanced glycation end-products and circulating DNA in patients with acute pancreatitis. Pancreatology.

[143] Zhao B., Chen Y., Sun W.W., Chen W.W., Ma L., Yang Z.T., Huang J., Chen E.Z., Fei J., Mao E.Q. (2016). Effect of S100A12 and soluble receptor for advanced glycation end products on the occurrence of severe acute pancreatitis. J. Dig. Dis..

[144] Bopp C., Hofer S., Weitz J., Bierhaus A., Nawroth P.P., Martin E., Büchler M.W., Weigand M.A. (2008). sRAGE is elevated in septic patients and associated with patients outcome. J. Surg. Res..

[145] Sadik N.A., Mohamed W.A., Ahmed M.I. (2012). The association of receptor of advanced glycated end products and inflammatory mediators contributes to endothelial dysfunction in a prospective study of acute kidney injury patients with sepsis. Mol. Cell Biochem..

[146] Narvaez-Rivera R.M., Rendon A., Salinas-Carmona M.C., Rosas-Taraco A.G. (2012). Soluble RAGE as a severity marker in community acquired pneumonia associated sepsis. BMC Infect. Dis..

[147] Achouiti A., Föll D., Vogl T., van Till J.W., Laterre P.F., Dugernier T., Wittebole X., Boermeester M.A., Roth J., van der Poll T. (2013). S100A12 and soluble receptor for advanced glycation end products levels during human severe sepsis. Shock.

[148] Brodska H., Malickova K., Valenta J., Fabio A., Drabek T. (2013). Soluble receptor for advanced glycation end products predicts 28-day mortality in critically ill patients with sepsis. Scand. J. Clin. Lab. Investig..

[149] Hamasaki M.Y., Barbeiro H.V., de Souza H.P., Machado M.C., da Silva F.P. (2014). sRAGE in septic shock: A potential biomarker of mortality. Rev. Bras. Ter. Intensiv..

[150] Matsumoto H., Matsumoto N., Ogura H., Shimazaki J., Yamakawa K., Yamamoto K., Shimazu T. (2015). The clinical significance of circulating soluble RAGE in patients with severe sepsis. J. Trauma Acute Care Surg..

[151] Hofer S., Uhle F., Fleming T., Hell C., Schmoch T., Bruckner T., Weigand M.A., Brenner T. (2016). RAGE-mediated inflammation in patients with septic shock. J. Surg. Res..

[152] Meertens J.H., Nienhuis H.L., Lefrandt J.D., Schalkwijk C.G., Nyyssönen K., Ligtenberg J.J., Smit A.J., Zijlstra J.G., Mulder D.J. (2016). The Course of Skin and Serum Biomarkers of Advanced Glycation Endproducts and Its Association with Oxidative Stress, Inflammation, Disease Severity, and Mortality during ICU Admission in Critically Ill Patients: Results from a Prospective Pilot Study. PLoS ONE.

[153] Pranal T., Pereira B., Berthelin P., Roszyk L., Godet T., Chabanne R., Eisenmann N., Lautrette A., Belville C., Blondonnet R. (2018). Clinical and Biological Predictors of Plasma Levels of Soluble RAGE in Critically Ill Patients: Secondary Analysis of a Prospective Multicenter Observational Study. Dis. Markers.

[154] Rodriguez-Ruiz E., Lopez-Lago A., Hernandez-Vaquero R., Granja-Gomez I., Estany-Gestal A., Alvarez E., Garcia-Gonzalez M., Garcia-Allut J.L. (2020). First-Days Reduction of Plasma and Skin Advanced Glycation End Products is Related to Outcome in Septic Patients. Shock.

[155] Yang P., Iffrig E., Harris F., Holder A.L., Martin G.S., Esper A.M. (2022). Serial Measurements of Protein Biomarkers in Sepsis-Induced Acute Respiratory Distress Syndrome. Crit. Care Explor..

[156] Galasko D., Bell J., Mancuso J.Y., Kupiec J.W., Sabbagh M.N., van Dyck C., Thomas R.G., Aisen P.S. (2014). Clinical trial of an inhibitor of RAGE-Aβ interactions in Alzheimer disease. Neurology.

[157] Li J., Wang K., Huang B., Li R., Wang X., Zhang H., Tang H., Chen X. (2021). The receptor for advanced glycation end products mediates dysfunction of airway epithelial barrier in a lipopolysaccharides-induced murine acute lung injury model. Int. Immunopharmacol..

[158] Sabbagh M.N., Agro A., Bell J., Aisen P.S., Schweizer E., Galasko D. (2011). PF-04494700, an oral inhibitor of receptor for advanced glycation end products (RAGE), in Alzheimer disease. Alzheimer Dis. Assoc. Disord..

[159] He F., Gu L., Cai N., Ni J., Liu Y., Zhang Q., Wu C. (2022). The HMGB1-RAGE axis induces apoptosis in acute respiratory distress syndrome through PERK/eIF2α/ATF4-mediated endoplasmic reticulum stress. Inflamm. Res..

[160] Mi L., Zhang Y., Xu Y., Zheng X., Zhang X., Wang Z., Xue M., Jin X. (2019). HMGB1/RAGE pro-inflammatory axis promotes vascular endothelial cell apoptosis in limb ischemia/reperfusion injury. Biomed. Pharmacother..

[161] He D.W., Liu D.Z., Luo X.Z., Chen C.B., Lu C.H., Na N., Huang F. (2024). HMGB1-RAGE axis contributes to myocardial ischemia/reperfusion injury via regulation of cardiomyocyte autophagy and apoptosis in diabetic mice. Biol. Chem..

[162] Zhang L., Jiang Y., Deng S., Mo Y., Huang Y., Li W., Ge C., Ren X., Zhang H., Zhang X. (2021). S100B/RAGE/Ceramide signaling pathway is involved in sepsis-associated encephalopathy. Life Sci..

[163] Lutterloh E.C., Opal S.M., Pittman D.D., Keith J.C., Tan X.Y., Clancy B.M., Palmer H., Milarski K., Sun Y., Palardy J.E. (2007). Inhibition of the RAGE products increases survival in experimental models of severe sepsis and systemic infection. Crit. Care.

[164] Kuhla A., Norden J., Abshagen K., Menger M.D., Vollmar B. (2013). RAGE blockade and hepatic microcirculation in experimental endotoxaemic liver failure. Br. J. Surg..

[165] Huttunen H.J., Fages C., Kuja-Panula J., Ridley A.J., Rauvala H. (2002). Receptor for advanced glycation end products-binding COOH-terminal motif of amphoterin inhibits invasive migration and metastasis. Cancer Res..

[166] Lee S., Piao C., Kim G., Kim J.Y., Choi E., Lee M. (2018). Production and application of HMGB1 derived recombinant RAGE-antagonist peptide for anti-inflammatory therapy in acute lung injury. Eur. J. Pharm. Sci..

[167] Zhang J., Xu X., Rao N.V., Argyle B., McCoard L., Rusho W.J., Kennedy T.P., Prestwich G.D., Krueger G. (2011). Novel sulfated polysaccharides disrupt cathelicidins, inhibit RAGE and reduce cutaneous inflammation in a mouse model of rosacea. PLoS ONE.

[168] Arumugam T., Ramachandran V., Gomez S.B., Schmidt A.M., Logsdon C.D. (2012). S100P-derived RAGE antagonistic peptide reduces tumor growth and metastasis. Clin. Cancer Res..

[169] Han Y.T., Choi G.I., Son D., Kim N.J., Yun H., Lee S., Chang D.J., Hong H.S., Kim H., Ha H.J. (2012). Ligand-based design, synthesis, and biological evaluation of 2-aminopyrimidines, a novel series of receptor for advanced glycation end products (RAGE) inhibitors. J. Med. Chem..

[170] Han Y.T., Kim K., Choi G.I., An H., Son D., Kim H., Ha H.J., Son J.H., Chung S.J., Park H.J. (2014). Pyrazole-5-carboxamides, novel inhibitors of receptor for advanced glycation end products (RAGE). Eur. J. Med. Chem..

[171] Zheng J., Zhu W., He F., Li Z., Cai N., Wang H.H. (2021). An Aptamer-Based Antagonist against the Receptor for Advanced Glycation End-Products (RAGE) Blocks Development of Colorectal Cancer. Mediat. Inflamm..

[172] Manigrasso M.B., Pan J., Rai V., Zhang J., Reverdatto S., Quadri N., DeVita R.J., Ramasamy R., Shekhtman A., Schmidt A.M. (2016). Small Molecule Inhibition of Ligand-Stimulated RAGE-DIAPH1 Signal Transduction. Sci. Rep..

[173] Faruqui T., Singh G., Khan S., Khan M.S., Akhter Y. (2023). Differential gene expression analysis of RAGE-S100A6 complex for target selection and the design of novel inhibitors for anticancer drug discovery. J. Cell Biochem..

[174] Burstein A.H., Sabbagh M., Andrews R., Valcarce C., Dunn I., Altstiel L. (2018). Development of Azeliragon, an Oral Small Molecule Antagonist of the Receptor for Advanced Glycation Endproducts, for the Potential Slowing of Loss of Cognition in Mild Alzheimer’s Disease. J. Prev. Alzheimer’s Dis..

[175] Kostura M.J., Kindy M.S., Burstein A., Valcarce C., Polisetti D., Andrews R., Mjalli A.M. (2014). P3-032: Efficacy of RAGE Antagonists in Murine Model of Alzheimer’s Disease. Alzheimer’s Dement..

[176] Burstein A.H., Grimes I., Galasko D.R., Aisen P.S., Sabbagh M., Mjalli A.M. (2014). Effect of TTP488 in patients with mild to moderate Alzheimer’s disease. BMC Neurol..

[177] Liu A., Zhang W., Wang S., Wang Y., Hong J. (2020). HMGB-1/RAGE signaling inhibition by dioscin attenuates hippocampal neuron damage induced by oxygen-glucose deprivation/reperfusion. Exp. Ther. Med..

[178] Suchal K., Malik S., Khan S.I., Malhotra R.K., Goyal S.N., Bhatia J., Kumari S., Ojha S., Arya D.S. (2017). Protective effect of mangiferin on myocardial ischemia-reperfusion injury in streptozotocin-induced diabetic rats: Role of AGE-RAGE/MAPK pathways. Sci. Rep..

[179] Suchal K., Malik S., Khan S.I., Malhotra R.K., Goyal S.N., Bhatia J., Ojha S., Arya D.S. (2017). Molecular Pathways Involved in the Amelioration of Myocardial Injury in Diabetic Rats by Kaempferol. Int. J. Mol. Sci..

[180] Rani N., Arya D.S. (2024). Modulation of PPAR-γ/Nrf2 and AGE/RAGE signaling contributes to the chrysin cardioprotection against myocardial damage following ischemia/reperfusion in diabetic rats. J. Pharm. Pharmacol..

[181] Ghoneim M.E., Abdallah D.M., Shebl A.M., El-Abhar H.S. (2020). The interrupted cross-talk of inflammatory and oxidative stress trajectories signifies the effect of artesunate against hepatic ischemia/reperfusion-induced inflammasomopathy. Toxicol. Appl. Pharmacol..

[182] Younis N.S., Abdelnaby R.M., Mohamed M.E. (2023). Hepatoprotective effects of linalool against liver ischemia-reperfusion: The role of Nrf2/HO-1/NQO1 and TLR4/RAGE/NFκB pathways. Eur. Rev. Med. Pharmacol. Sci..

[183] Zhang Z.H., Yang H.X., Jin Q., Wu Y.L., Cui Z.Y., Shang Y., Liu J., Zhan Z.Y., Lian L.H., Nan J.X. (2021). Luteolin attenuates hepatic injury in septic mice by regulating P2X7R-based HMGB1 release. Food Funct..

[184] Zhao F., Fang Y., Deng S., Li X., Zhou Y., Gong Y., Zhu H., Wang W. (2017). Glycyrrhizin Protects Rats from Sepsis by Blocking HMGB1 Signaling. Biomed. Res. Int..

[185] Tamada K., Nakajima S., Ogawa N., Inada M., Shibasaki H., Sato A., Takasawa R., Yoshimori A., Suzuki Y., Watanabe N. (2019). Papaverine identified as an inhibitor of high mobility group box 1/receptor for advanced glycation end-products interaction suppresses high mobility group box 1-mediated inflammatory responses. Biochem. Biophys. Res. Commun..

[186] Solmaz V., Kaya M., Uslu F.B., Atasoy O., Erbaş O. (2020). Papaverine Has Therapeutic Potential for Sepsis-Induced Neuropathy in Rats, Possibly via the Modulation of HMGB1-RAGE Axis and Its Antioxidant Prosperities. J. Investig. Surg..

[187] Özkul B., Sever İ.H., Yiğittürk G., Elgörmüş Ç.S., Gür S.G., Erbaş O. (2023). Demonstration of ameliorating effect of papaverine in sepsis-induced acute lung injury on rat model through radiology and histology. Ulus. Travma Acil Cerrahi Derneği.

[188] Shi J., Xu H., Cavagnaro M.J., Li X., Fang J. (2021). Blocking HMGB1/RAGE Signaling by Berberine Alleviates A1 Astrocyte and Attenuates Sepsis-Associated Encephalopathy. Front. Pharmacol..

[189] He J., Qin W., Jiang S., Lin Y., Lin Y., Yang R., Xu M., Liu Q. (2024). Oxymatrine attenuates sepsis-induced inflammation and organ injury via inhibition of HMGB1/RAGE/NF-κB signaling pathway. Drug Dev. Res..

[190] Qin Y., Li W., Liu J., Wang F., Zhou W., Xiao L., Zhou P., Wu F., Chen X., Xu S. (2024). Andrographolide ameliorates sepsis-induced acute lung injury by promoting autophagy in alveolar macrophages via the RAGE/PI3K/AKT/mTOR pathway. Int. Immunopharmacol..

[191] Bu Y., Li Z., Wang C., Yu Y., Liu C., Sun Y., Sun Z., Gong W., Luo J., Yue Z. (2025). Anemoside B4 targets RAGE to attenuate ferroptosis in sepsis-induced acute lung injury. Front. Pharmacol..

[192] Coimbra M., Isacchi B., van Bloois L., Torano J.S., Ket A., Wu X., Broere F., Metselaar J.M., Rijcken C.J., Storm G. (2011). Improving solubility and chemical stability of natural compounds for medicinal use by incorporation into liposomes. Int. J. Pharm..

[193] Jain H., Chella N. (2021). Methods to improve the solubility of therapeutical natural products: A review. Environ. Chem. Lett..

[194] Ji M., Long L., Xiong S., Liu Z., Luo J., Liu D. (2025). Nanocrystalline Drug Delivery Systems for Natural Compounds: Progress, Challenges and Future Opportunities. Int. J. Nanomed..

